# Next-Generation Intestinal Toxicity Model of Human Embryonic Stem Cell-Derived Enterocyte-Like Cells

**DOI:** 10.3389/fvets.2021.587659

**Published:** 2021-09-16

**Authors:** Bokyeong Ryu, Mi-Young Son, Kwang Bo Jung, Ukjin Kim, Jin Kim, Ohman Kwon, Ye Seul Son, Cho-Rok Jung, Jae-Hak Park, C-Yoon Kim

**Affiliations:** ^1^Department of Laboratory Animal Medicine, College of Veterinary Medicine, Seoul National University, Seoul, South Korea; ^2^Stem Cell Convergence Research Center, Korea Research Institute of Bioscience and Biotechnology (KRIBB), Daejeon, South Korea; ^3^Department of Functional Genomics, Korea Research Institute of Bioscience and Biotechnology School of Bioscience, Korea University of Science and Technology, Daejeon, South Korea; ^4^Gene Therapy Research Unit, Korea Research Institute of Bioscience and Biotechnology, Daejeon, South Korea; ^5^Department of Veterinary Physiology, College of Veterinary Medicine, Konkuk University, Seoul, South Korea

**Keywords:** alternative testing method, embryonic stem cell, enterotoxicity, intestinal toxicity, toxicology

## Abstract

The gastrointestinal tract is the most common exposure route of xenobiotics, and intestinal toxicity can result in systemic toxicity in most cases. It is important to develop intestinal toxicity assays mimicking the human system; thus, stem cells are rapidly being developed as new paradigms of toxicity assessment. In this study, we established human embryonic stem cell (hESC)-derived enterocyte-like cells (ELCs) and compared them to existing *in vivo* and *in vitro* models. We found that hESC-ELCs and the *in vivo* model showed transcriptomically similar expression patterns of a total of 10,020 genes than the commercialized cell lines. Besides, we treated the hESC-ELCs, *in vivo* rats, Caco-2 cells, and Hutu-80 cells with quarter log units of lethal dose 50 or lethal concentration 50 of eight drugs—chloramphenicol, cycloheximide, cytarabine, diclofenac, fluorouracil, indomethacin, methotrexate, and oxytetracycline—and then subsequently analyzed the biomolecular markers and morphological changes. While the four models showed similar tendencies in general toxicological reaction, hESC-ELCs showed a stronger correlation with the *in vivo* model than the immortalized cell lines. These results indicate that hESC-ELCs can serve as a next-generation intestinal toxicity model.

## Introduction

There is a need to develop models for intestinal toxicity assessment. Despite the gastrointestinal tract (GIT) being one of the body's defense lines, proper models for evaluating toxicity and predicting the effects of chemicals in orally administrated cases are not available. The oral route is one of the most common forms of exposure to drugs and chemicals above inhalation and skin absorption (1). Orally administered substances are first metabolized by the GIT (2), and the parental materials and their metabolites are absorbed through the GIT (3). During these metabolic and absorption processes, the original parental substances or their metabolites may cause damage to the GIT (4, 5). Loss of the intestinal barrier function *via* toxic reactions can ultimately result in the degradation of systemic defenses (6–9). Since intestinal toxicity may result in systemic toxicity, screening of potential intestinal toxicants may result in the identification of substances that cause systemic toxicity. Therefore, among the newly developed or currently used substances, it is necessary to eliminate the substances that may have toxicological effects on the GIT.

For intestinal toxicity assessment, human intestinal slides are not easily accessible to most researchers despite having the correct three-dimensional architecture and physiological complexity (10). The currently available models, other than the human intestinal slides, include *in vivo* rodent models and *in vitro* cell lines. Rodents have been widely used (11), and they can show systemic responses in both specific and related organs; thus, an appropriate animal model would make possible predicting drug behavior in the human body (12). However, they differ from humans in aspects of the species both physiologically and biochemically; their anatomical structures are different, including the morphological structure of the stomach and the shape of the small intestinal villi (13). Their metabolisms are also different in terms of the expressions and catalytic activities of xenobiotic-metabolizing enzymes such as cytochrome P450 (CYP) (14–16). In addition to the scientific reasons, the use of animals is not only costly but has also been criticized from an ethical perspective based on the 3R principle of replacement, reduction, and refinement (17). Therefore, a new model is needed as a suitable alternative to laboratory animals mimicking *in vivo* rodent responses to intestinal toxicants.

Cell culture-based models may be useful as alternatives to *in vivo* rodents by improving predictability typically resulting from species differences and minimizing ethical concerns. Commercially available immortalized cell line models such as Caco-2 and Hutu-80 can be used to observe the responses of human-derived intestinal cells in screening for toxic substances. Additionally, these cells are relatively convenient to maintain under general experimental conditions and can be used for high-throughput approaches (18). However, conventional cell culture models cannot be used to predict organ-specific toxicity because reproducing the *in vivo* structure and physiological conditions using cells is difficult (19, 20).

Stem cells can be used to prepare organ-like models (21–23) based on their pluripotency (24, 25). Human embryonic stem cells (hESCs) can be used to develop a functional *in vitro* intestinal model to complement existing laboratory animals and immortalized cell lines. hESCs have been reported to differentiate into enterocytes, which can closely mimic the mature intestinal epithelium while showing comparable expression levels of mature intestinal markers and increased intestine-specific functional activities (26). For the present study, even though the three-dimensional organoids have been developed as a powerful tool for *in vitro* intestinal research, we used a re-seeded two-dimensional model to present an appropriate *in vitro* high-throughput screening model (27). We compared the hESC-derived enterocyte-like cells (hESC-ELCs) with the following models: rats as a representative laboratory animal model and Caco-2 and Hutu-80 cells as representative commercialized cell models. Subsequently, we compared the toxicological responses within these models under treatment of eight representative intestinal toxicants. The toxicants were chosen based on their clinical reports, which include antibiotics, non-steroidal anti-inflammatory drugs, and antineoplastic drugs. They were used to determine the utility of hESC-ELCs as an intestinal toxicity evaluation model.

## Materials and Methods

### Drugs and Chemicals

Bovine serum albumin (BSA), chloramphenicol (CHL; CAS no. 56-75-7, product no. C0857), cycloheximide (CHX; CAS no. 66-81-9, product no. 1810), cytarabine (Ara-C; CAS no. 147-94-4, product no. PHR1787), diclofenac (DIC; CAS no. 15307-79-6, product no. D6899), dimethyl sulfoxide (DMSO), fluorouracil (5-FU; CAS no. 51-21-8, product no. F6627), indomethacin (INDO; CAS no. 53-86-1, product no. I7378), methotrexate (MTX; CAS no. 59-05-2, product no. A6770), MTT [3-(4,5-dimethylthiazol-2-yl)-2,5-diphenyltetrazolium bromide] (CAS no. 298-93-1, product no. 92 M2128), oxytetracycline (OTC; CAS no. 2058-46-0, product no. O5875), and paraformaldehyde were obtained from Sigma-Aldrich (St. Louis, MO, USA). Fetal bovine serum (FBS) and phosphate-buffered saline (PBS) were obtained from Gibco (Grand Island, NY, USA), and Dulbecco's modified Eagle's medium/Nutrient Mixture F-12 (DMEM/F-12), low-glucose DMEM, knockout serum replacement, non-essential amino acids, GlutaMAX™, β-mercaptoethanol, B27, N2, and L-glutamine were obtained from Thermo Fisher Scientific (Waltham, MA, USA). Basic fibroblast growth factor, Activin A, FGF4, Wnt3a, and epidermal growth factor were obtained from R&D Systems (Minneapolis, MN, USA).

### Animal Experiments

All animal experiments were performed according to the guidelines for the care and use of laboratory animals approved by the Institutional Animal Care and Use Committee, Seoul National University. Permission to use rats was granted by the Institutional Animal Care and Use Committee, Seoul National University (Permission No. SNU-170310-3-1).

Forty-five healthy male Sprague–Dawley rats aged 6 weeks were obtained from Orient Bio Co. (Gyeonggi-do, South Korea) and maintained under an artificial 12-h light/dark cycle at a constant temperature of 22 ± 1°C and humidity of 55 ± 10%. The rats were housed individually in cages for 1 week for acclimatization to the laboratory conditions. Rats aged 7 weeks with a mean ± standard deviation (SD) body weight of 257.5 ± 19.84 g were used in this study, following the OECD TG 423 recommendations for toxicity testing. Forty-five rats were randomly divided into nine groups: a control (CONT) group and the following eight treatment groups: CHL, CHX, Ara-C, DIC, 5-FU, INDO, MTX, and OTC.

### Cell Culture

Caco-2 (ATCC^®^ HTB-37, KCLB lot no. 26877) and Hutu-80 (ATCC^®^ 114 HTB-40, KCLB lot no. 30040) were obtained from the Korean Cell Line Bank. Although the Caco-2 cells were derived from human colonic adenocarcinoma, they were chosen as a worldwide gold standard of *in vitro* intestinal model (28, 29). Hutu-80 cells were chosen as they were derived from the human duodenum. The Caco-2 cells were maintained in DMEM/F-12 medium containing 10% FBS and differentiated as described previously (30). The Hutu-80 cells were maintained in low-glucose DMEM medium containing 10% FBS. These commercialized cells were used at near 100% confluence for comparison with the hESC-ELCs as similarly as possible. The cells were cultured at 37°C with 5% CO_2_.

The H9 hESC line (WiCell Research Institute, Madison, WI, USA) was cultured as described previously (26, 31). Briefly, hESCs were maintained on γ-irradiated mouse embryonic fibroblasts in hESC medium containing 80% DMEM/F-12 medium, 20% knockout serum replacement, 1% non-essential amino acids, 1% GlutaMAX™, 55 μM β-mercaptoethanol, and 8 ng/ml basic fibroblast growth factor.

The hESC-ELCs were generated from hESCs as described previously (32). Briefly, hESCs were differentiated into definitive endoderm (DE) by treatment with 100 ng/ml Activin A for 3 days and then further differentiated into hindgut (HG) with 250 ng/ml FGF4 and 50 ng/ml Wnt3a treatment for 4 days. HG cells were dissociated into single cells and re-seeded for differentiation into hESC-ELCs in DMEM/F-12 containing 2% FBS, 2% B27, 1% N2, 2 mM L-glutamine, 1% non-essential amino acid, and 20 ng/ml epidermal growth factor (differentiation medium). The culture media were replaced with fresh differentiation medium every other day and passaged every 7 days.

The hESCs and hESC-ELCs were monitored by quantitative real-time polymerase chain reaction (qPCR) and immunofluorescence analyses to characterize the cells. To prepare total RNA and complementary DNA (cDNA) synthesis of the cells, the RNeasy kit (Qiagen, Hilden, Germany) and Superscript IV cDNA synthesis kit (Thermo Fisher Scientific) were used according to the manufacturers' protocol. qPCR was performed with a 7500 Fast Real-time PCR System (Applied Biosystems, Foster City, CA, USA). Information for the primers used in this study is listed in Supplementary Table 1.

To conduct the immunofluorescence analysis, hESCc and hESC-ELCs were fixed with 4% paraformaldehyde for 15 min. The fixed cells were permeabilized with 0.1% Triton X-100 before blocking with 4% BSA. Samples were incubated with primary antibodies (Supplementary Table 2) diluted in 4% BSA at 4°C overnight, and then the secondary antibodies were treated for 1 h at room temperature. DAPI staining was performed for visualization of nuclei. To mount the slides, a DAKO Fluorescent Mounting Medium (DAKO, Carpinteria, CA, USA) was used. The samples were examined with an FV1000 Live confocal microscope (Olympus, Tokyo, Japan). After confirmation of differentiation, the hESC-ELCs were used for RNA sequencing and drug-induced toxicity analysis.

### RNA Extraction and Sequencing

RNA sequencing (RNA-seq) was conducted to compare the basic gene expression levels in untreated naive rat small intestine and hESC-ELCs. To perform RNA-seq, total RNA was extracted from the entire layers of the proximal part of the small intestine (5 cm from pylorus) and the cells using GeneAll^®^ Hybrid-R™ (Seoul, South Korea) according to the manufacturer's protocol. All RNA samples were determined as of high and comparable quality by the Agilent 2100 Bioanalyzer System (Böblingen, Germany). cDNA libraries were generated according to standard procedures using the Lexogen QuantSeq 3′ mRNA-Seq Library Prep Kit (Vienna, Austria). The libraries were sequenced on an Illumina NextSeq500 (San Diego, CA, USA) in single-end (SE) 75-base mode. Each set of RNA-seq data was derived from a single biological sample.

The Illumina reads were trimmed to only retain reads with values higher than the average Q20 in Phred quality score using BBDuk (part of BBtools). The remaining reads were mapped to the respective reference genome sequences (hg19 for human cells and rn6 for the rat intestinal tissue; genome database: University of California, Santa Cruz) using Bowtie2 (33). Calculation reads were counted using Bedtools (https://bedtools.readthedocs.io/en/latest/). Read mapping and expression quantification were performed separately for each sample.

From the National Center for Biotechnology Information Gene Expression Omnibus (NCBI GEO), the RNA-seq data of H9 hESCs (GEO no. GSE118106), differentiated Caco-2 cells (GEO no. GSE97977), Hutu-80 cells (GEO no. GSE59857), human fetal small intestine (GEO no. GSE108369), and human adult small intestine (GEO no. GSE117875) were obtained. To compare the gene expression data derived from human and rat, each homolog gene was matched from two species using the Ensemble Genes 95 database (GRCh38.p12). The gene expression levels were then quantile normalized using edgeR (34). All genes were compared to determine the similarity of hESC-ELCs to the *in vivo* model.

### Toxicity Tests

To compare the *in vivo* rat model and the *in vitro* Caco-2 cells, Hutu-80 cells, and hESC-ELCs, the dosages in each model were calculated based on lethal dose 50 (LD_50_) *in vivo* and lethal concentration 50 (LC_50_) *in vitro*. Each LD_50_ for rat oral administration was referred to the literature, and each LC_50_ in Caco-2 cells, Hutu-80 cells, and hESC-ELCs at 24 h was analyzed. Cell viabilities were determined by the MTT assay. The LD_50_ and LC_50_ values of the drugs are shown in Supplementary Table 3 and in Supplementary Figures 1–3 for Caco-2 cells, Hutu-80 cells, and hESC-ELCs, respectively.

Rats in the treatment groups were administered a single oral dose of a quarter unit of LD_50_ (LD_50_/10^0.25^, *n* = 5), while rats in the CONT group were administered the vehicle consisting only of 0.9% normal saline (*n* = 5). The cells were exposed to each drug at a quarter unit of the LC_50_ (LC_50_/10^0.25^), while the control received naive serum-free media as a vehicle (*n* = 3), considering the clinical human dose. The conditions of the drug administration are shown in Supplementary Table 3. The animals were sacrificed after 24 h treatment in a CO_2_ chamber, and the entire layers of the proximal part of the small intestine (5 cm from pylorus) were excised for total RNA extraction and histopathological examination. The cells were examined morphologically after 24 h treatment and then harvested for total RNA extraction.

### MTT Assay for LC_50_ Measurement

The MTT assay for the determination of the LC_50_ values was conducted as previously described (35). Briefly, 7 × 10^4^ cells per well were seeded into 96-well plates and exposed to various concentrations of all drugs for 24 h. All media were removed and the cells were incubated with 200 μl of 0.5 mg/ml MTT solution and dissolved in PBS at 37°C for 4 h. Removal of 100 μl of the MTT solution was followed by the addition of 100 μl of DMSO to each well, and the plates were gently shaken for 10 min to achieve complete dissolution. Aliquots of the resulting solution were transferred into new 96-well plates and the absorbance recorded at 570 nm using an Epoch Microplate Spectrophotometer (Bio-Tek, Winooski, VT, USA).

### qPCR Analysis

To perform qPCR of the drug-treated samples from *in vivo* rats, Caco-2 cells, and hESC-ELCs, the extracted RNA from each treated sample was reverse transcribed using products from Enzynomics (Daejeon, South Korea) to obtain cDNA according to the manufacturer's protocol. Briefly, 1 μg of RNA and 1 μl of 100 μM random hexamer were mixed and incubated in a T100™ Thermal Cycler (Bio-Rad, Hercules, CA, USA) for 5 min at 70°C and then placed on ice. Two microliters of 10X M-MLV RT buffer, 1 μl of M-MLV reverse transcriptase (200 U/μl), 2 μl of dNTP mixture (2 mM each), 0.5 μl of RNase inhibitor, and sterile water up to 20 μl were added to each sample, and the samples were then incubated in the T100™ Thermal Cycler using the following protocol: 10 min at 25°C, 60 min at 42°C, 5 min at 94°C, and holding at 4°C.

qPCR was performed with a qPCR System CFX Connect™ (Bio-Rad) and CFX Manager 3.1.1517.0823 software (Bio-Rad) using TOPreal™ qPCR 2X PreMIX (SYBR Green with low ROX; Enzynomics) according to the manufacturer's protocol. Briefly, qPCR was performed in a final volume of 20 μl containing 1 μl cDNA, 1 μl forward primer and 1 μl reverse primer, 10 μl TOPreal™ qPCR 2X PreMIX, and sterile water up to 20 μl. The PCR program consisted of an initial denaturing cycle at 95°C for 15 min, followed by 40 amplification cycles of 10 s at 95°C, 15 s at the melting temperature, and 30 s at 72°C.

The primers were purchased from Macrogen (Seoul, South Korea) and their sequences listed in Supplementary Table 4. Briefly, *CYP1A2* (rat *Cyp1a2*), *CYP2B6* (rat *Cyp2b1*), *CYP2C8* (rat *Cyp2c7*), *CYP2C9* (rat *Cyp2c11*), *CYP2C19* (rat *Cyp2c6v1*), *CYP2D6* (rat *Cyp2d3*), *CYP2E1* (rat *Cyp2e1*), *CYP3A4* (rat *Cyp3a2*), *CYP24A1* (rat *Cyp24a1*), *CES2, MAOA*, and *NAT* were chosen as the metabolism-related genes. Additionally, *Catalase, PXR, SOD1, GPx1, HO1*, and *iNOS* oxidative stress indicators; *Bad, Bax, Bcl-2, Bcl-XL, Bid, Casp3, Casp7, Casp8, Casp9, Fas, PUMA, TGF-*β*1, p53*, and *APC* as apoptosis-related genes; *IL-*β*1, IL1RN, IL-6, NF-*κ*B, TNF, TLR2*, and *TLR4* as inflammation-related genes; and *OCLN, CLDN1, CLDN3, VIL1*, and *TJP1* as tight junction structure-related genes were used. Each sample was measured in triplicate. The results were normalized to the level of *ACTB*, which was used as an internal control (ΔCt = Ct_targetgene_ – Ct_ACTB_, with Ct as the cycle threshold). Relative fold changes in the expressions of the target genes were determined using the comparative 2^−ΔΔCt^ method, with ΔΔCt = ΔCt_treatedsample_ – ΔCt_control_ (36).

### Histopathological Analysis

Intestinal tissue samples obtained at the point 5 cm from the pylorus were assayed. For histopathological evaluation with a light microscope, intestinal tissues were freshly excised and fixed in 10% neutralized formalin. The tissues were then processed by routine tissue techniques using a Tissue-Tek^®^ VIP™ 5 Jr Tissue Processor (SAKURA, Staufen, Germany) and embedded in paraffin using HistoCore Arcadia (Leica, Wetzlar, Germany). Paraffin-embedded specimens were cut into 4-μm-thick sections. The sections were mounted on slides and stained with hematoxylin and eosin (H&E). The slides were examined to detect any morphological changes in the tissue using an Olympus PROVIS AX70 light microscope (Tokyo, Japan), Nikon DS-Ri2 camera (Tokyo, Japan), and NIS-Elements BR 4.50.00 software (Tokyo, Japan).

Besides, to detect apoptosis, TUNEL (terminal deoxynucleotidyl transferase-mediated dUTP nick-end labeling) staining was conducted on the rat intestinal tissues and the cells according to the manufacturer's instructions (Hoffmann-La Roche, Basel, Switzerland). All fluorescent images were analyzed with the Ti-2000 fluorescence microscope (Nikon, Minato-ku, Tokyo, Japan), and the apoptotic indices were calculated based on TUNEL-positive nuclei and total nuclei.

### Statistical Analysis

All statistical analyses were carried out using GraphPad Prism 5.01 software (GraphPad Software, Inc., La Jolla, CA, USA), R 3.5.2 (The R Foundation for Statistical Computing c/o Institute for Statistics and Mathematics, Vienna, Austria), ClueGO v2.5.5 and CluePedia v1.5.5 on Cytoscape v3.7.2 (Cytoscape Consortium, San Diego, CA, USA) on Java script v1.8.0_162 (Oracle Corporation; Santa Clara, CA, USA) (37), TIGR MultiExperiment Viewer (MeV) 4.9.0 (The Institute for Genomic Research, and ArrayAssist software, Stratagene; http://www.tm4.org/mev) (38), and the Database for Annotation, Visualization and Integrated Discovery (DAVID) v6.8 (39, 40).

To identify the genes with significant differential expressions in qPCR, one-way analysis of variance and Dunnett's multiple comparison test were performed using GraphPad Prism software. A *p* < 0.05 was considered to indicate significance.

Gene Ontology (GO) network and Enrichment Pathway analysis for hESC-ELCs against H9 hESCs (GEO no. GSE118106) was performed using ClueGO. Upregulated differentially expressed genes (DEGs) in the differentiated hESC-ELCs were selected based on fold change 128 times higher than that of H9 hESCs. ClueGo analysis incorporated GO for biological process (EBI, UniProt-GOA). The pathway's restriction was set to *p* < 0.05, and a GO tree interval of 4–6 was used to specify the GO terms. The connectivity score (kappa score) was set to 0.4.

Principal component analysis (PCA) was performed to visualize and quantify multidimensional variations between hESC-ELCs and the other models. Principal components were calculated using the function “prcomp” and function “autoplot(clara)” found in the R statistical programming language and plotted using the R package.

PCA also was performed for the RNA-seq results under naive states and the qPCR results in the toxicity test. Additionally, hierarchical clustering of the genes to visualize the expression patterns was performed using Pearson's correlation and average linkage after log2 transformation in TIGR MeV. Clustering was conducted for comparison in the RNA-seq data and the toxicity test.

A Venn diagram was drawn to visualize the intersection between the upregulated DEGs of hESC-ELCs and those of the other models against H9 hESCs (GEO no. GSE118106). The elements of intersections were counted using the function “vennCounts” found in the R statistical programming language and plotted using Gliffy (Perforce Software, Inc., Minneapolis, MN, USA). The intersections were analyzed using ClueGO and DAVID.

Additionally, correlations were assessed between the *in vitro* models and the *in vivo* model in the toxicity test using Pearson's correlation coefficient (*r*) after testing for normality. To determine the strength of the associations, the correlation coefficient *r* was interpreted as follows: 0.90–1.00 or from −0.90 to −1.00, very highly correlated; 0.70–0.90 or from −0.70 to −0.90, highly correlated; 0.50–0.70 or from −0.50 to −0.70, moderately correlated; 0.30–0.50 or from −0.30 to −0.50, lowly correlated; and 0.00–0.30 or from 0.00 to −0.30, negligibly correlated (41). Significance was assigned to differences with *p* < 0.05.

## Results

### Differentiation of Human Enterocytes From hESCs

**Figure 1:**
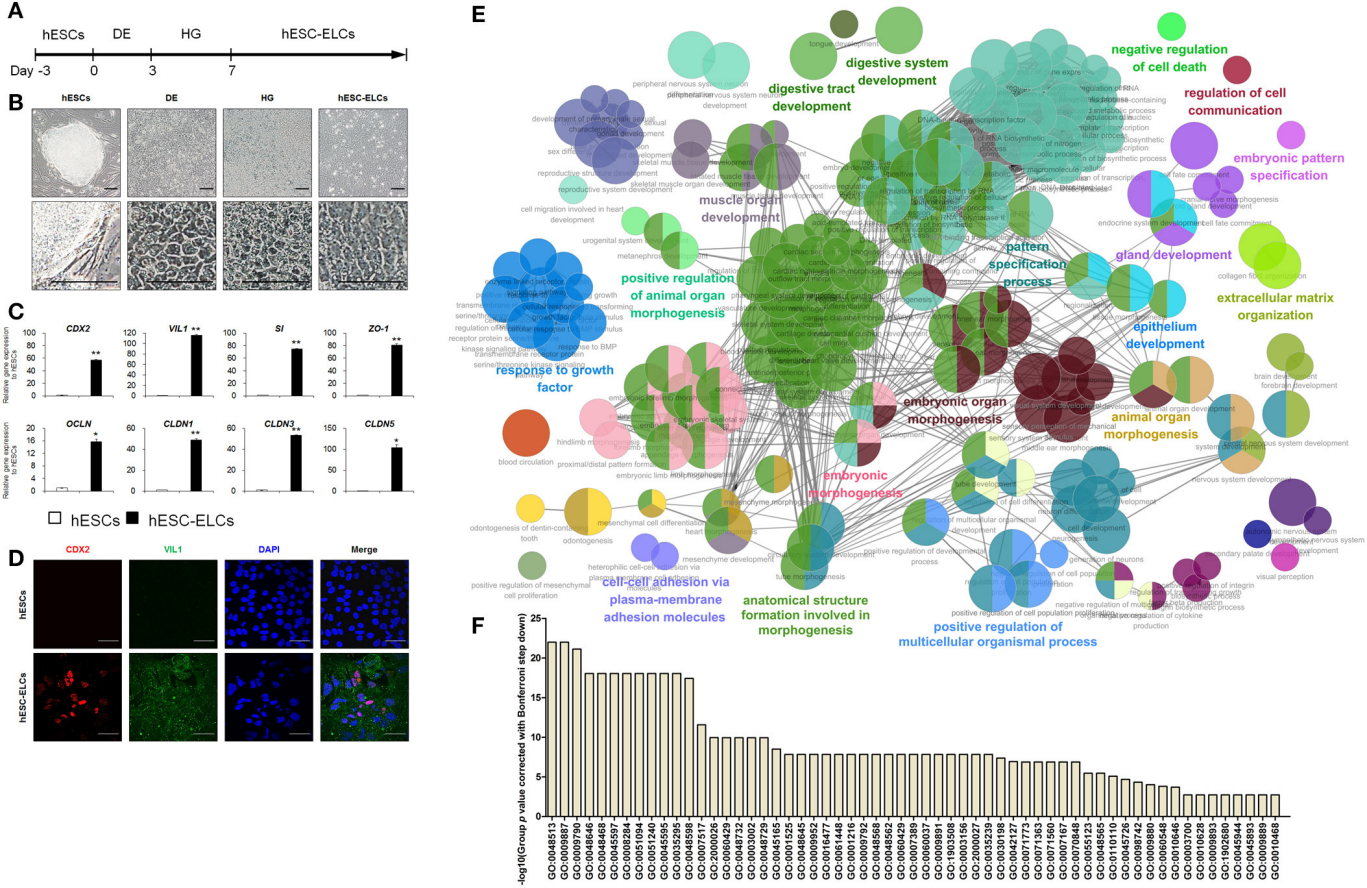
Differentiation of human embryonic stem cells into human enterocytes. **(A)** Scheme of the differentiation protocol of human embryonic stem cell-derived enterocyte-like cells (hESC-ELCs) from hESCs. **(B)** Representative morphological changes during differentiation from hESCs to definitive endoderm (DE), hindgut (HG), and hESC-ELCs. *Scale bars*, 100 μm. **(C)** Expression levels of the intestine-specific marker (*CDX2*), enterocyte markers (*VIL1* and *SI*), and the tight junction markers (*ZO-1, OCLN, CLDN1, CLDN3*, and *CLDN5*) in hESCs and hESC-ELCs assessed by qPCR analysis. Data are presented as the mean ± SEM. *p < 0.05; **p < 0.01. **(D)** Immunofluorescent staining of hESCs and hESC-ELCs with the intestine-specific marker (*CDX2*) and enterocyte marker (*VIL1*). *Scale bars*, 20 μm. **(E)** Gene Ontology (GO) network and Enrichment Pathway analysis for the hESC-ELCs assessed by ClueGO. Upregulated differentially expressed genes (DEGs; fold change > 128) in hESC-ELCs compared to H9 hESCs were analyzed. **(F)** The GOs of DEGs in **(E)** were presented with their *p* values.

To differentiate hESC-ELCs from hESCs, we conducted a differentiation protocol (Figure 1A) that slightly modified the previously reported protocol (32). hESCs were efficiently differentiated into DE, HG, and hESC-ELCs, as indicated by their altered morphologies (Figure 1B). The hESC-ELCs showed epithelial morphologies such as cuboidal shapes and arrangement along the lines. Next, we examined the efficiency of hESC-ELC differentiation based on the expressions of the intestinal enterocyte-specific markers, such as the intestinal transcription factor *CDX2*, the enterocyte markers *VIL1* and *SI*, and the tight junction markers *ZO-1, OCLN, CLDN1, CLDN3*, and *CLDN5*, which were significantly enhanced in hESC-ELCs compared to that in hESCs (Figure 1C). We also observed enhanced expressions of the enterocyte marker proteins CDX2 and VIL1 in hESC-ELCs by immunofluorescence analysis (Figure 1D).

When analyzing the upregulated DEGs of hESC-ELCs compared to those of hESCs (Figure 1E), the enriched pathways included mainly GO:0055123 (digestive system development), GO:0048565 (digestive tract development), GO:0060429 (epithelium development), GO:0030198 (extracellular matrix organization), GO:0048732 (gland development), GO:0007517 (muscle organ development), GO:0007389 (pattern specification process), GO:0110110 (positive regulation of animal organ morphogenesis), GO:0051240 (positive regulation of multicellular organismal process), GO:0048646 (anatomical structure formation involved in morphogenesis), GO:0009887 (animal organ morphogenesis), GO:0048598 (embryonic morphogenesis), GO:0048562 (embryonic organ morphogenesis), GO:0009880 (embryonic pattern specification), GO:0098742 (cell–cell adhesion *via* plasma membrane adhesion molecules), GO:0060548 (negative regulation of cell death), GO:0010646 (regulation of cell communication), and GO:0070848 (response to growth factor) (Figure 1F). These enriched GO networks indicated the embryonic development into tube-shaped multicellular organs including epithelial cells, extracellular matrix, muscular layers, and glandular function. These results demonstrate that hESCs effectively differentiated into hESC-ELCs, as revealed by the marked increases in the expressions of the intestinal enterocyte-specific marker genes and proteins.

### Comparison of hESC-ELCs With *in vivo* and *in vitro* Models

**Figure 2:**
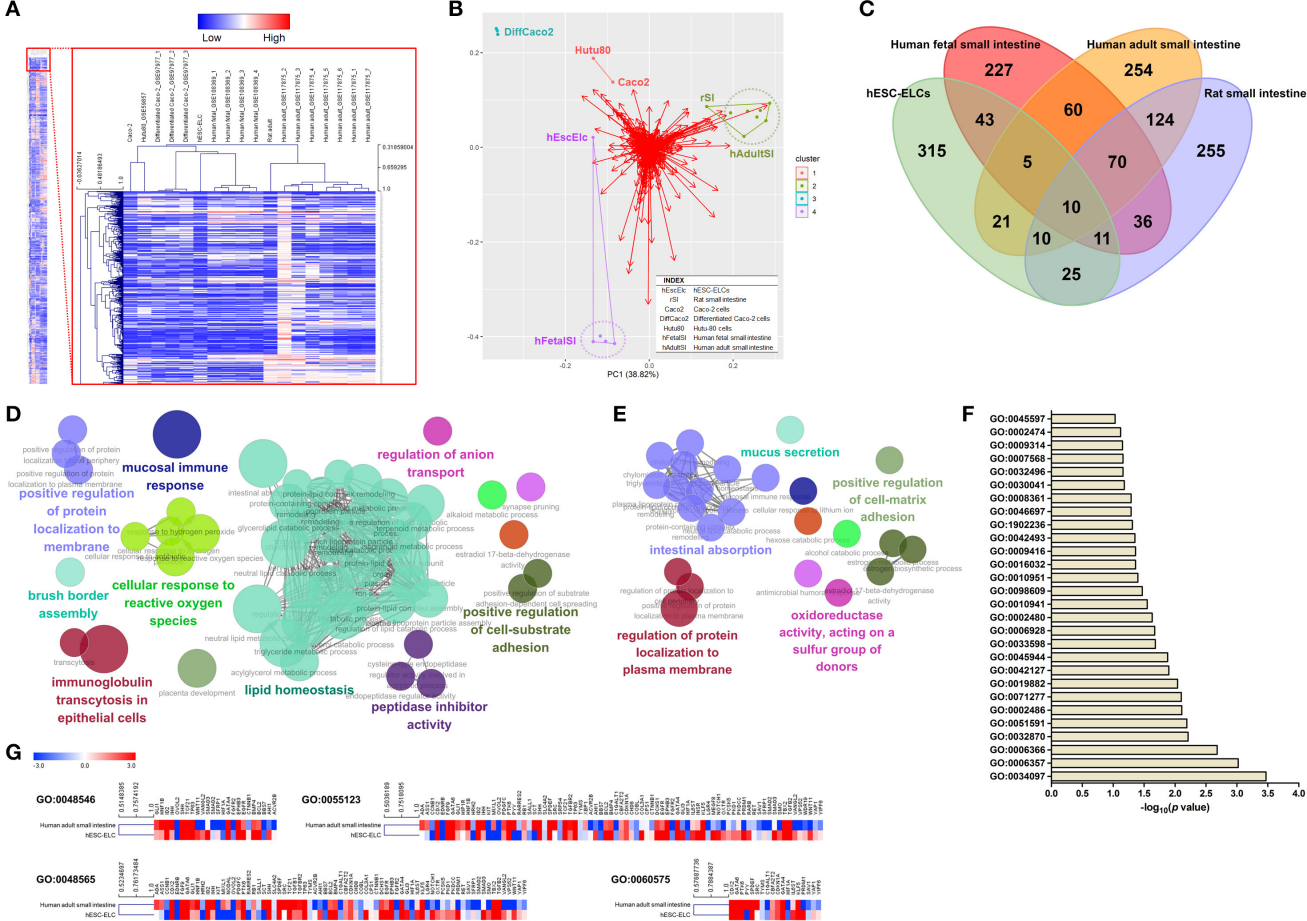
Transcriptomic assessment of human embryonic stem cell-derived enterocyte-like cells (hESC-ELCs) and other small intestine models. hESC-ELCs, rat small intestine, Caco-2 cells, Hutu-80 cells, and human adult and fetal small intestines without any treatment were used for RNA sequencing (RNA-seq). **(A)** Heatmap of a total number of 10,020 genes used to cluster the models hierarchically. The heatmap represents six models (*columns*) and their genes (*rows*). The *color scale at the top* represents the expression level, where *red, blue*, and *white* indicate upregulation, downregulation, and an unaltered expression, respectively. **(B)** Relative distances of the seven models visualized using principal component analysis (PCA). *Dots with colors* represent each model, and the *red arrows* are for each gene in the transcriptomic analysis. Clustered samples share the *colors and lines*. *hEscElc*, hESC-ELCs; *rSI*, rat small intestine; *Caco2*, Caco-2 cells; *Hutu80*, Hutu-80 cells; *hFetalSI*, human fetal small intestine; *hAdultSI*, human adult small intestine. **(C)** Venn diagram visualizing the intersection between hESC-ELCs and the *in vivo* models. The intersection of the human and rat adult small intestines has 214 genes in common. **(D)** The enriched biological pathways were assessed by ClueGO. The intersection of the human adult and fetal small 698 intestines has 145 genes in common. **(E)** The enriched pathways were also assessed. The intersection of hESC-ELCs and the human adult small intestines has 46 genes in common. **(F)** The enriched top 28 pathways were assessed by DAVID. **(G)** Comparison of the hESC-ELCs and human adult small intestines in intestine-related biological pathways.

Gene expressions in hESC-ELCs, Caco-2 cells, Hutu-80 cells, rat small intestine, human adult small intestine, and human fetal small intestine were compared and a heatmap was drawn showing the expression levels of a total of 10,020 genes (Figure 2A). The hESC-ELCs were clustered with a human fetal model before clustered with the adult intestinal models (human adult small intestine and rat small intestine) and especially with the *in vitro* models (Caco-2 and Hutu-80 cells). This dendrogram would mean a stronger correlation with the hESC-ELCs and the young *in vivo* model than that of other *in vitro* or adult models in naive conditions. The correlation was visualized in PCA (purple color in Figure 2B), and the hESC-ELCs showed a closer position with the young *in vivo* model.

When compared to hESCs, the upregulated DEGs were visualized in a Venn diagram of the hESC-ELCs and other intestinal samples (Figure 2C). The hESC-ELCs and human adult small intestine show an intersection including 46 genes, while two adult models (human and rat adult small intestines) have one including 214 genes and two human models (human adult and fetal small intestines) have one including 145 genes. The intersection of two adult models shows enriched biological pathways such as GO:1904970 (brush border assembly), GO:0034614 (cellular response to reactive oxygen species), GO:0002414 (immunoglobulin transcytosis in epithelial cells), GO:0055088 (lipid homeostasis), GO:0002385 (mucosal immune response), GO:0030414 (peptidase inhibitor activity), GO:0010811 (positive regulation of cell substrate adhesion), GO:1905477 (positive regulation of protein localization to membrane), and GO:0044070 (regulation of anion transport) (Figure 2D), indicating mature functions of the small intestine. Additionally, the intersection of the human adult and fetal models shows enriched pathways such as GO:0001954 (positive regulation of cell matrix adhesion), GO:0070254 (mucus secretion), GO:0016667 (oxidoreductase activity, acting on a sulfur group of donors), GO:1903076 (regulation of protein localization to plasma membrane), and GO:0050892 (intestinal absorption) (Figure 2E), indicating basic functions of the small intestine. Meanwhile, the intersection of hESC-ELCs and the human adult model has enriched pathways, with the top 28 including GO:0045597 (positive regulation of cell differentiation), GO:0008361 (regulation of cell size), and GO:0030041 (actin filament polymerization) (Figure 2F), indicating cellular differentiation from stem cells.

For the intestine-related biological pathways such as GO:0048546 (digestive tract morphogenesis), GO:0048565 (digestive tract development), GO:0055123 (digestive system development), and GO:0060575 (intestinal epithelial cell differentiation), the hESC-ELCs and the human adult small intestine show over 0.50 of Pearson's correlation coefficient (*r*) (Figure 2G), which means a moderately strong correlation between them. Fold changes of the hESC-ELCs and the adult model compared to hESCs were used.

### Relative Changes in mRNA Expression Under Treatment of Intestinal Toxicants

**Figure 3:**
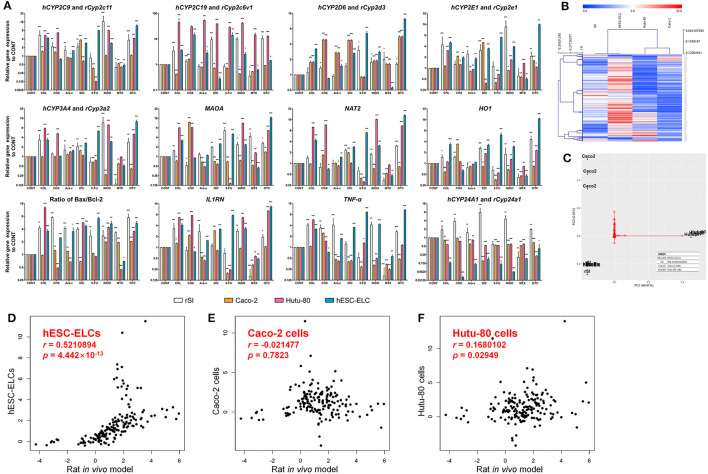
Relative mRNA expression levels in rat small intestines, Caco-2 cells, Hutu-80 cells, and human embryonic stem cell-derived enterocyte-like cells (hESC-ELCs). Rat small intestines, Caco-2 cells, Hutu-80 cells, and hESC-ELCs were treated with chloramphenicol (CHL), cycloheximide (CHX), cytarabine (Ara-C), diclofenac (DIC), fluorouracil (5-FU), indomethacin (INDO), methotrexate (MTX), oxytetracycline (OTC), or vehicle and assessed by quantitative PCR (qPCR). **(A)** The relative expressions of *hCYP2C9/rCyp2c11, hCYP2C19/rCyp2c6v1, hCYP2D6/yCyp2d3, hCYP2E1/rCyp2e1, hCYP3A4/rCyp3a2, MAOA, NAT2, HO1, IL1RN, TNF*, and *hCYP24A1/rCyp24a1* were monitored. Also, the ratio of *Bax*/*Bcl-2* was calculated. Data shown are the mean ± SEM. *p < 0.05; **p < 0.01; ***p < 0.001. **(B)** Heatmap of the entire set of genes represented by four models (*columns*) and their genes treated with each drug (*rows*). The *color scale at the top* represents the expression level, where *red, blue*, and *white* indicate upregulation, downregulation, and an unaltered expression, respectively. **(C)** Relative distances of the four models visualized using principal component analysis (PCA). *Black dots* represent each model, and the *red arrows* are for each gene in the qPCR analysis. *hEscElc*, hESC-ELCs; *rSI*, rat small intestine; *Caco2*, Caco-2 cells; *Hutu80*, Hutu-80 cells. **(D–F)** Correlations with the *in vivo* model under treatment of intestinal toxicants of hESC-ELCs **(D)**, Caco-2 cells **(E)**, and Hutu-80 cells **(F)** (for all panels, the *x*-axis shows the rat *in vivo* gene expression and the *y*-axis shows the cell gene expression). The strength of the correlations between the *in vitro* models and the *in vivo* model is shown as Pearson's correlation coefficient (*r*) with associated *p*-values.

To assess the general responses to drug-induced toxicity, the transcript levels of the metabolism-, oxidative stress-, apoptosis-, inflammation-, and tight junction structure-related genes were monitored in the hESC-ELCs, Caco-2 cells, Hutu-80 cells, and *in vivo* rats. As shown in Figure 3A, the relative expression levels of the metabolism-related genes, such as *CYP2C9* (rat *Cyp2c11*), *CYP2C19* (rat *Cyp2c6v1*), *CYP2D6* (rat *Cyp2d3*), *CYP2E1* (rat *Cyp2e1*), *CYP3A4* (rat *Cyp3a2*), *MAOA*, and *NAT2*, were generally increased in the rat intestines and hESC-ELCs, except in the 5-FU- and MTX-treated groups. The relative expression of the *HO1* gene as an oxidative stress-related gene showed similar changes in the rat intestines and hESC-ELCs, and the ratio of *Bax/Bcl-2* as an indicator of apoptosis increased in all groups. Additionally, the relative expressions of the inflammation-related genes such as *IL1RN* and *TNF-*α were similarly changed in the rat intestines and hESC-ELCs. In contrast, the relative expression of *CYP24A1* (rat *Cyp24a1*) decreased in all groups of Caco-2 cells, Hutu-80 cells, and hESC-ELCs and increased in the rat intestines treated with CHL, CHX, Ara-c, DIC, INDO, and OTC.

The general tendencies of the results were visualized in a heatmap (Figure 3B). While the general tendencies were similar in the rat intestines, Caco-2 cells, Hutu-80 cells, and hESC-ELCs, the overall results determined in the analysis show that hESC-ELCs have shorter dendrites with the *in vivo* model than with the *in vitro* Caco-2 cells and Hutu-80 cells. These relative distances were also proven in a PCA (Figure 3C). The hESC-ELCs positioned closer to the *in vivo* model than to the *in vitro* models.

To quantitatively determine the correlations between the *in vivo* model and the *in vitro* models including Caco-2 cells, Hutu-80 cells, and hESC-ELCs, the Pearson's correlation coefficient (*r*) was calculated within the qPCR results. The *r* value between the rat intestines and hESC-ELCs was 0.5210894, indicating a moderately strong correlation (Figure 3D; *x*-axis: rat *in vivo* gene expression, *y*-axis: hESC-ELC gene expression), while the *r* value between the rat intestines and Caco-2 cells was −0.021477, indicating a negligible correlation (Figure 3E; *x*-axis: rat *in vivo* gene expression, *y*-axis: Caco-2 cell gene expression), and that between the rat intestines and Hutu-80 cells was 0.1680102, also indicating a negligible correlation (Figure 3F; *x*-axis: rat *in vivo* gene expression, *y*-axis: Hutu-80 cell gene expression).

The means, standard deviations, and interquartile ranges for the correlation calculations are presented in Table 1. The *in vivo* model and the hESC-ELCs were significantly correlated (*p* = 4.442 × 10^−13^), while the Caco-2 cells (*p* = 0.7823) and Hutu-80 cells (*p* = 0.02949) were not.

**Table 1:** Means, standard deviations, and interquartile ranges of Pearson's correlation coefficient (*r*) for qPCR analysis in each model.

	**Mean**	**SD**	**IQR**	**0%**	**25%**	**50%**	**75%**	**100%**
Rat *in vivo*	1.252	1.633	1.803	−4.248	0.223	1.088	2.026	5.955
Caco-2 cells	1.208	1.729	1.978	−4.248	0.143	1.036	2.121	11.501
Hutu-80 cells	1.418	2.144	2.123	−4.248	0.115	1.01	2.238	14.035
hESC-ELCs	1.637	1.902	1.872	−3.11	0.145	0.978	2.017	11.501

*hESC-ELCs, human embryonic stem cell-derived enterocyte-like cells*.

The overall results demonstrate that the general metabolic and toxic responses of hESC-ELC following exposure to intestinal toxicants are more similar to those in the *in vivo* rat intestines than to those in Caco-2 cells or Hutu-80 cells despite the expression differences in some genes.

### Morphological Changes Under Treatment of Intestinal Toxicants

**Figure 4:**
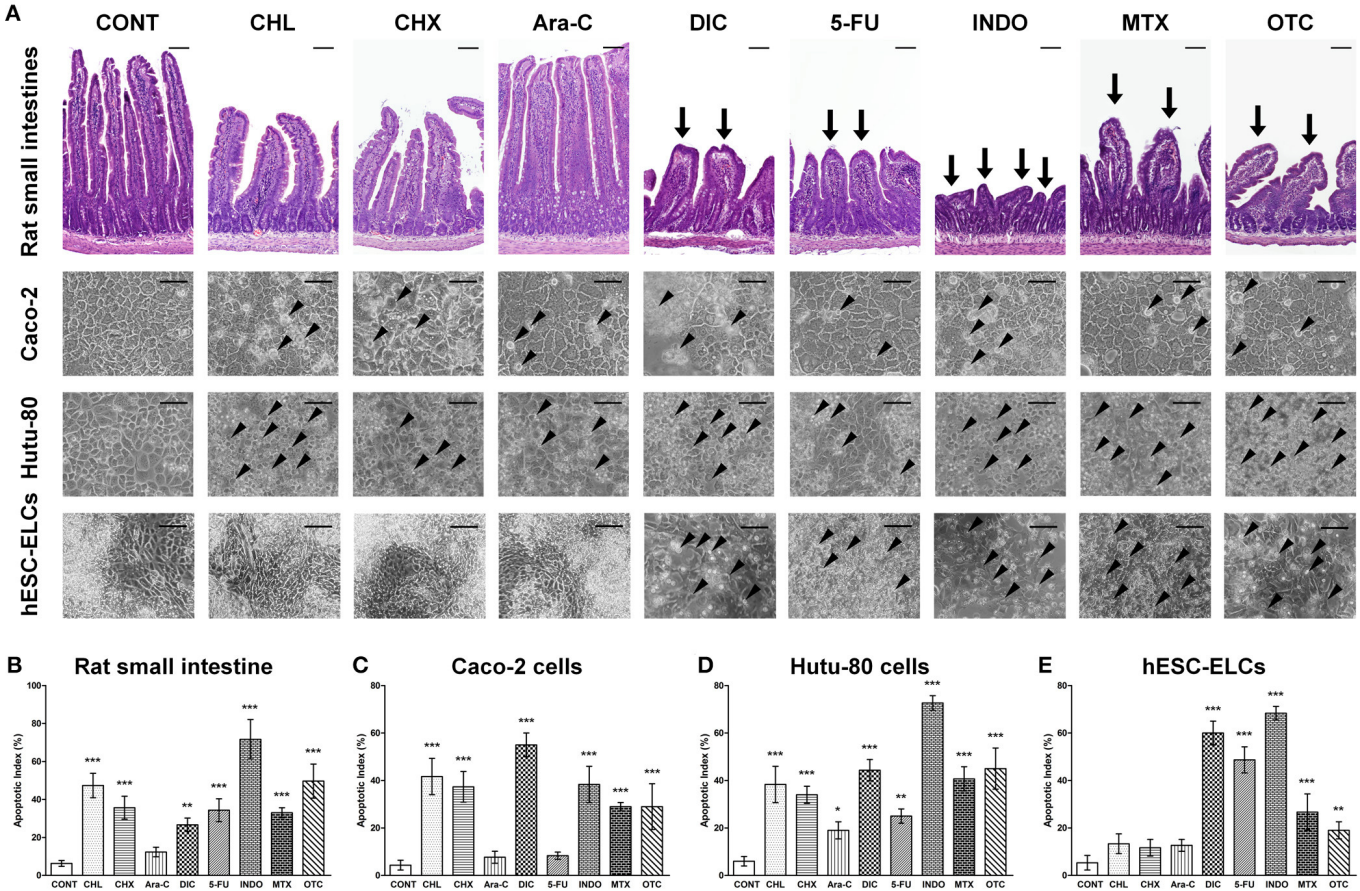
Representative morphological changes in human embryonic stem cell-derived enterocyte-like cells (hESC-ELCs) and in the other models under treatment of intestinal toxicants. Rat small intestines, Caco-2 cells, Hutu-80 cells, and hESC-ELCs were treated with chloramphenicol (CHL), cycloheximide (CHX), cytarabine (Ara-C), diclofenac (DIC), fluorouracil (5-FU), indomethacin (INDO), methotrexate (MTX), oxytetracycline (OTC), or vehicle and the morphological changes were analyzed. **(A)** The rat tissues were stained with hematoxylin and eosin (HandE), and the rat tissues and cells were assessed. *Arrows* indicate the damaged intestinal architectures in the rat intestines, and *arrowheads* indicate the characteristics of apoptotic cell death in Caco-2 cells, Hutu-80 cells, and hESC-ELCs. All *scale bars*, 100 μm. **(B–E)** Apoptotic indices were also calculated in rat small intestines **(B)**, Caco-2 cells **(C)**, Hutu-80 cells **(D)**, and hESC-ELCs **(E)**. Data shown are the mean ± SD. *p < 0.05; **p < 0.01; ***p < 0.001.

Following treatments with the drugs, the morphological changes in each group were observed by microscopy to confirm damages, which are monitored molecularly. As shown in Figure 4A, rat small intestine tissues exhibited damaged architectures with decreased villus height and blunted villi, consistent with *in vitro* cellular damages such as cell shrinkage and the detachment of cells from the cell culture substratum for Caco-2 cells, Hutu-80 cells, and hESC-ELCs. Apoptosis was confirmed using the TUNEL assay (Supplementary Figure 4), and the apoptotic indices were calculated in the *in vivo* model, Caco-2 cells, Hutu-80 cells, and hESC-ELCs (Figures 4B–E, respectively). In all models, the structural changes and the apoptotic indices were most severe in the INDO-treated groups, followed by the DIC-, 5-FU-, and the OTC-treated groups. The CHL-, CHX-, and MTX-treated groups showed mild changes, while the Ara-c-treated groups exhibited subtle changes in all the models.

## Discussion

Stem cells can be developed into toxicological models for various organs because of their pluripotency (42). While commercialized cell lines lack the ability to mimic *in vivo* reactions and it is difficult to predict systemic reactions in the human body, which is required in tests related to metabolism or toxicity (43), stem cell-based models can complement these conventional models. Additionally, models derived from stem cells can be utilized not only in drug development but also in disease modeling. Therefore, stem cells may be developed as next-generation models for researches. In this study, hESC-ELCs were efficiently obtained from hESCs and showed significant small intestine-like features compared to hESCs. The small intestine-like features were proven morphologically, molecular biologically, and transcriptomically.

Additionally, they showed similarities to the *in vivo* small intestines in a total of 10,020 genes as naive states. Common genes of the differentially upregulated genes in hESC-ELCs and the *in vivo* small intestine were analyzed to include intestinal differentiation-related pathways. These tendencies could be found with a comparison of the gene expressions in the small intestine-related GO terms, in which the hESC-ELCs showed a moderately strong correlation with the human adult small intestine. Besides, under treatment of representative intestinal toxicants, the hESC-ELCs and the *in vivo* model showed similar patterns in the gene expression changes. Particularly, the similarities were visualized and quantified using a heatmap, a PCA, and Pearson's correlation coefficient *r* values. Compared to *in vivo* rats in terms of the toxicological responses, the hESC-ELCs (|*r*| = 0.5210894) were found to be significantly more similar than are Caco-2 cells (|*r*| = 0.021477) or Hutu-80 cells (|*r*| = 0.1680102), while the four models showed similar tendencies in general toxicological reactions molecular biologically and morphologically.

Notably, the hESC-ELCs exhibited a similar pattern to the *in vivo* model of the small intestine in terms of genes, which are selected as an intersection of the human database and the rat database, within naive states and states under treatment of toxic substances. The correlations within RNA-seq would support the existence of the phenotypic correlations (44). Aside from the analysis in the naive states, metabolic processes in toxicological models were analyzed. Metabolism is one of the important intestinal functions because the severity of the toxic response may depend on the ability to produce metabolites (45); chemically reactive metabolites, in addition to the original material, may be toxic. In this study, the expressions of genes involved in metabolism increased following treatments with drugs such as DIC, 5-FU, and OTC in rats and hESC-ELCs. DIC is metabolized to produce metabolites such as 4-hydroxy diclofenac, 5-hydroxy diclofenac, diclofenac acyl glucuronide, or diclofenac glutathione thioester (46), OTC is metabolized to produce 4-epi-oxytetracycline (47), and 5-FU can produce a small amount of 5-fluorouridine 5′-triphosphate or 5-fluoro-2′-deoxyuridine 5′-triphosphate (48). These chemically active metabolites may affect the overall toxicity.

Among the metabolism-related factors, CYP is an important superfamily and includes heme-thiolate proteins that catalyze the biotransformation of drugs and other xenobiotics (49). For CYPs, those in enterocytes play an important role in the metabolism and excretion of the drugs, while hepatic P450 plays the most important role (50). For example, it has been reported that the intestine contributes to drug metabolism equivalently to hepatic CYP3A by first-pass metabolism (51–53), although the expression level of CYP3A in the intestine is ~1% of that observed in the liver (54). In the development of artificial enterocytes, CYP3A is the most abundant P450 in the human small intestine, followed by CYP2C9, CYP2C19, and CYP2D6 (49). These drug-metabolizing enzymes were reported to be expressed in the basal state in tissues without induction, and their expression levels were increased by exposure to xenobiotics (55). Thus, CYP expression should be considered when developing an intestinal toxicity model.

In the currently used commercial cell models, the expressions of CYPs do not mimic the *in vivo* patterns. In a typical culture environment, Caco-2 cells, which are derived from human colorectal adenocarcinoma and are being used worldwide as the gold standard of *in vitro* intestinal models, do not express substantial amounts of CYP3A4 (56), CYP2C9 (57), or CYP1A and express a small amount of CYP2D6 (58). In T84 cells, which are derived from human colorectal carcinoma and thought to be a potential alternative to Caco-2 cells (59), the expression of CYP3A4 is controversial (60), while expressions of CYP2B6, CYP2C9, CYP2C19, and CYP2E1 are lacking (61). Additionally, FHC cells, derived from the human colon, and HT29 cells, derived from human colorectal adenocarcinoma, cannot express CYP2B6, CYP2C9, CYP2C19, or CYP3A4 (61). Although Hutu-80 cells are derived from the small intestine, unlike in the large intestine cancer-derived cell lines, the expressions of CYP1A1, CYP1A2, CYP2C9/10, and CYP3A could not be detected also in Hutu-80 cells (62). Unlike commercialized cell models, hESC-ELCs can similarly reproduce *in vivo* metabolic functions, indicating that they can be developed as a toxicity assessment model.

During the drug-induced reactions, the expression of CYP24A1 was increased in the *in vivo* model and decreased in the two *in vitro* models; thus, the changes in Caco-2 cells, Hutu-80 cells, and hESC-ELCs coincided. While the expression of CYP24A1 can affect the degree of cell division (63), it may be decreased by an apoptosis mechanism initiated by oxidative stress (64). This phenomenon, in which the expression of CYP24A1 was not correlated in rats and hESC-ELCs, indicates that hESC-ELCs are limited as an *in vitro* model, like the conventional commercial cell lines such as Caco-2 cells and Hutu-80 cells. In contrast, differences in the general gene expression were observed between the rat intestines and the hESC-ELCs treated with anticancer drugs such as 5-FU and MTX. 5-FU has been reported to decrease the activities of CYPs (65), while other anticancer drugs increase these activities (66). Additionally, definitive differences between species were reported in anticancer drug metabolism (67–69). Considering the uncommon effects of anticancer drugs on CYPs and the species differences between humans and rats, the inconsistent results observed in the rat intestines and hESC-ELCs treated with 5-FU or MTX require further examination.

In conclusion, hESC-ELCs showed responses similar to those of an *in vivo* model, in contrast to Caco-2 cells and Hutu-80 cells, when untreated or treated with representative intestinal toxicants. Particularly, the gene expressions of the enzymes involved in metabolism had remarkably similar tendencies to the *in vivo* model, demonstrating that hESC-ELC cell lines may serve as a next-generation intestinal toxicity evaluation model.

## Data Availability Statement

The datasets presented in this study can be found in online repositories. The names of the repository/repositories and accession number(s) can be found below: NCBI BioProject Accession PRJNA649090.

## Ethics Statement

The animal study was reviewed and approved by The Institutional Animal Care and Use Committee of Seoul National University, Seoul 08826, Republic of Korea.

## Author Contributions

BR, M-YS, KJ, UK, JK, OK, YS, C-RJ, J-HP, and C-YK conceived and planned the experiments. BR, UK, and JK carried out the experiments. M-YS, KJ, OK, and YS contributed to sample preparation. BR, M-YS, KJ, UK, JK, OK, YS, C-RJ, J-HP, and C-YK contributed to the interpretation of the results. BR and C-YK took the lead in writing the manuscript. All authors provided critical feedback and helped guide the research, analysis, and manuscript.

## Funding

This research was supported by a grant from the KRIBB Research Initiative Program (grant number: KGM472212209710), Research Institute of Veterinary Science, College of Veterinary Medicine, Seoul National University, and BK21 PLUS Program for Creative Veterinary Science Research, College of Veterinary Medicine, Seoul National University.

## Conflict of Interest

The authors declare that the research was conducted in the absence of any commercial or financial relationships that could be construed as a potential conflict of interest.

## Publisher's Note

All claims expressed in this article are solely those of the authors and do not necessarily represent those of their affiliated organizations, or those of the publisher, the editors and the reviewers. Any product that may be evaluated in this article, or claim that may be made by its manufacturer, is not guaranteed or endorsed by the publisher.
