# Supplementary material for: Next-Generation Intestinal Toxicity Model of Human Embryonic Stem Cell-Derived Enterocyte-Like Cells
Source: Front Vet Sci. 2021 Sep 16;8:587659. doi: 10.3389/fvets.2021.587659 (PMC8481684; doi:10.3389/fvets.2021.587659)
Supplement: Supplementary file 2 [file Data_Sheet_1.PDF]

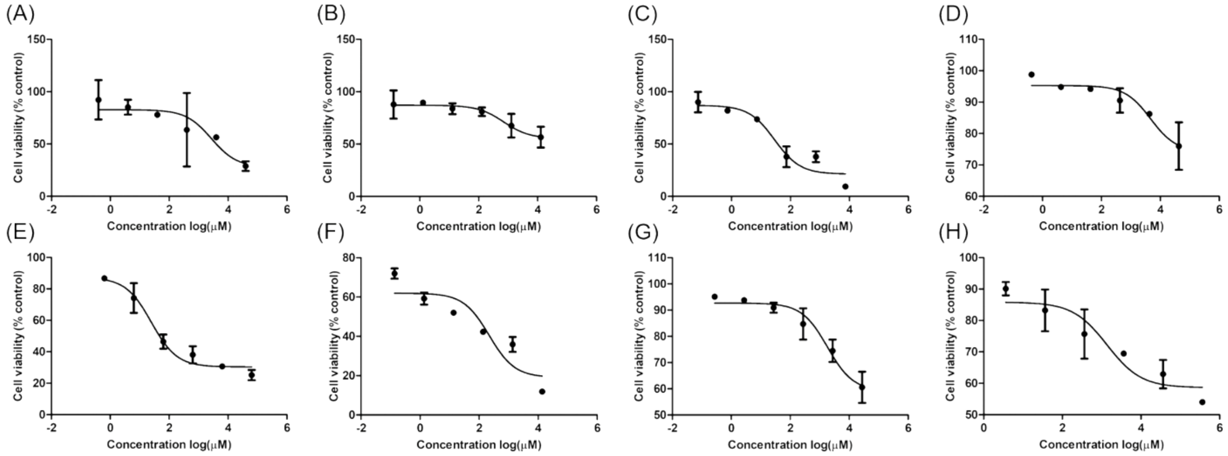

Figure S1. LC_{50} for 24 h of all drugs in Caco-2 cells.

Caco-2 cells were treated with six serial concentrations of eight drugs. Cell viabilities depend on logarithmic concentration of (A) CHL, (B) CHX, (C) Ara-C, (D) DIC, (E) 5-FU, (F) INDO, (G) MTX, and (H) OTC. Data shown are the mean ± SD.

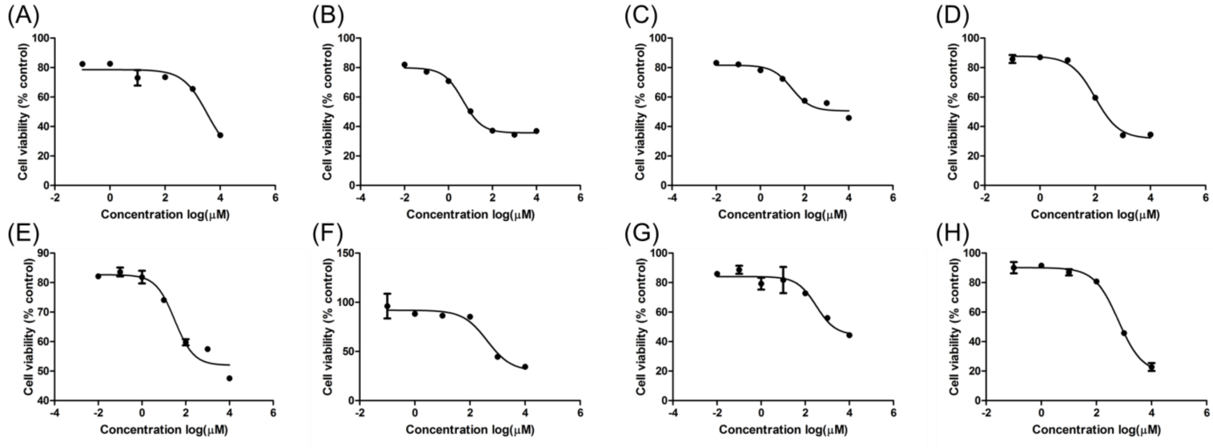

Figure S2. LC$_{50}$ for 24 h of all drugs in Hutu-80 cells.

Hutu-80 cells were treated with six serial concentrations of eight drugs. Cell viabilities depend on logarithmic concentration of (A) CHL, (B) CHX, (C) Ara-C, (D) DIC, (E) 5-FU, (F) INDO, (G) MTX, and (H) OTC. Data shown are mean ± SD.

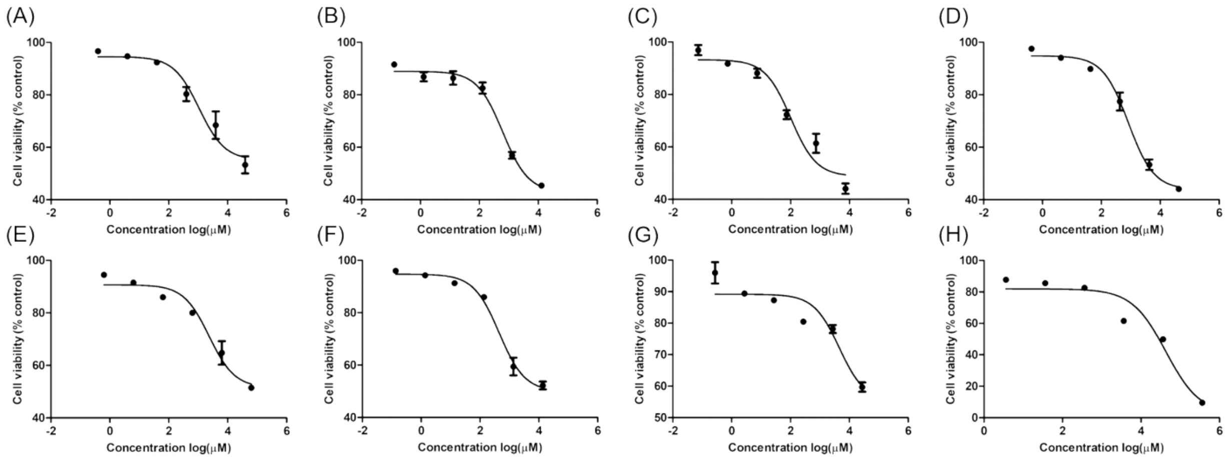

Figure S3. LC_{50} for 24 h of all drugs in hESC-ELCs.

hESC-ELCs were treated with six serial concentrations of eight drugs. Cell viabilities depend on logarithmic concentration of (A) CHL, (B) CHX, (C) Ara-C, (D) DIC, (E) 5-FU, (F) INDO, (G) MTX, and (H) OTC. Data shown are mean ± SD.

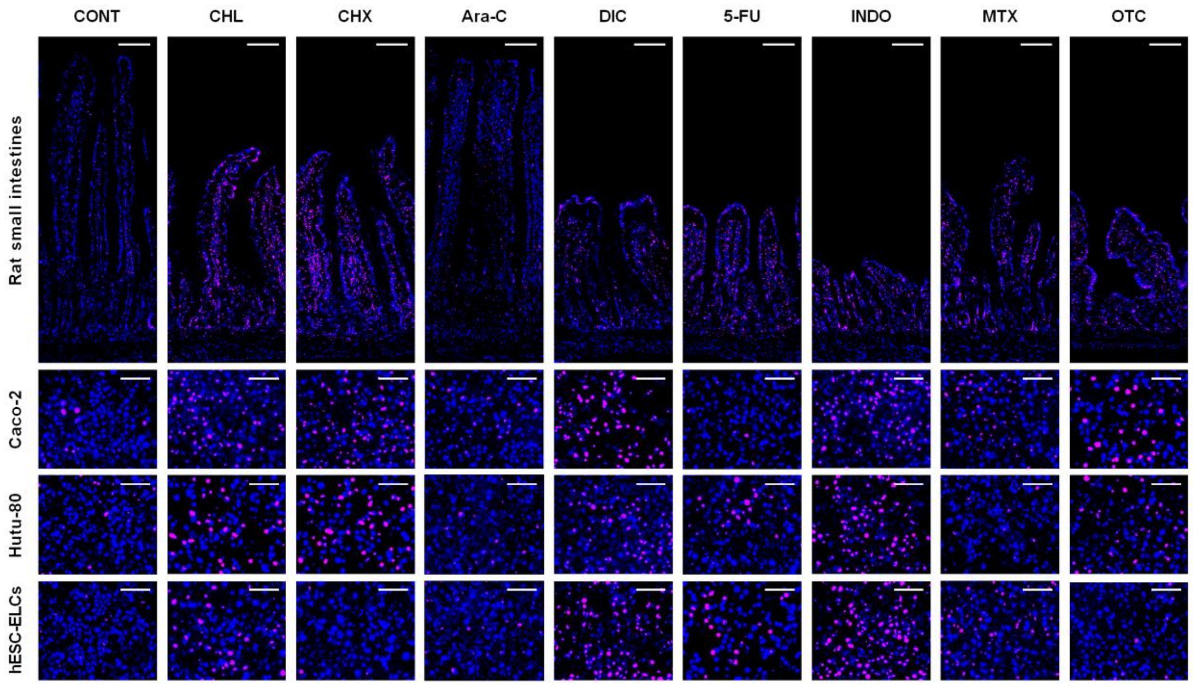

Figure S4. TUNEL-positive nuclei in rat small intestines, Caco-2 cells, Hutu-80 cells, and hESC-ELCs.

Rat small intestines, Caco-2 cells, Hutu-80 cells, and hESC-ELCs were treated with CHL, CHX, Ara-C, DIC, 5-FU, INDO, MTX, OTC, or vehicle and were stained with TUNEL (red) and DAPI (blue). Scale bars: 100 μm.

Table S1. Primers used for characterization of hESCs and hESC-ELCs.

Target gene	Primer (Forward)	Primer (Reverse)
GAPDH	GAAGGTGAAGGTCGGAGTC	GAAGATGGTGATGGGATTTC
CDX2	CTGGAGCTGGAGAAGGAGTTTC	ATTTTAACCTGCCTCTCAGAGAGC
VIL1	AGCCAGATCACTGCTGAGGT	TGGACAGGTGTTCCTCCTTC
SI	GGTAAGGAGAAACCGGGAAG	GCACGTCGACCTATGGAAAT
ZO-1	TGTGAGTCCTTCAGCTGTGGAA	GGAACTCAACACACCATTG
OCLN	CATTGCCATCTTTGCCTGTG	AGCCATAACCATAGCCATAGC
CLDN1	CCCAGTCAATGCCAGGTACG	GGGCCTTGGTGTTGGGTAAG
CLDN3	CAGGCTACGACCGCAAGGAC	GGTGGTGGTGGTGGTGTTGG
CLDN5	GCAGCCCCTGTGAAGATTGA	GTCTCTGGCAAAAAGCGGTG

Table S2. Antibodies used for characterization of hESCs and hESC-ELCs.

Antibodies	Catalog No.	Company	Dilution
anti-CDX2	ab15258	Abcam	1:100
anti-Villin1	sc-7672	Santa Cruz	1:50

Table S3. Oral LD_{50} in rats and LC_{50} in Caco-2 cells, Hutu-80 cells, and hESC-ELCs.

Drugs	CAS number	Rat per os (mg/kg)		Caco-2 cells (μM)	
		LD_{50}	LD_{50} /$10^{0.25}$	LC_{50}	LC_{50} /$10^{0.25}$
CHL	56-75-7	2500.00	1405.85	2710.19	1524.05
CHX	66-81-9	2.00	1.12	703.07	395.37
Ara-C	147-94-4	5000.00	2811.71	28.84	16.22
DIC	15307-86-5	53.00	29.80	4645.15	2612.16
5-FU	51-21-8	230.00	129.34	24.95	14.03
INDO	53-86-1	15.00	8.44	217.77	122.46
MTX	59-05-2	135.00	75.92	1823.90	1025.65
OTC	2058-46-0	5063.00	2847.13	1370.88	770.90

(Continued)

Drugs	Hutu-80 cells (μM)		hESC-ELCs (μM)	
	LC_{50}	LC_{50} /$10^{0.25}$	LC_{50}	LC_{50} /$10^{0.25}$
CHL	1081.43	608.14	3296.10	1853.53
CHX	613.76	345.14	4.54	2.56
Ara-C	101.16	56.89	25.94	14.59
DIC	824.14	463.45	98.86	55.59
5-FU	2349.63	1321.30	31.41	17.66
INDO	446.68	251.19	419.76	236.05
MTX	4560.37	2564.48	314.05	176.60
OTC	43151.91	24266.10	622.30	349.95

Table S4. Primer list of metabolism-, oxidative stress-, apoptosis-, inflammation-, and tight junction structure-related genes in human and rat.

Categories	Abbreviation	Gene	NCBI accession number	Primer (Forward)	Primer (Reverse)	Product size (bp)	Tm (°C)	Reference
**Human**								
Metabolism-related genes	*CYP1A2*	Cytochrome P450 family 1 subfamily A member 2	NM_000761.5	CTTCGCTACCTGCCTAACCC	GACTGTGTCAAATCCTGCTCC	243	60	(Ma et al., 2016)
	CYP2B6	Cytochrome P450 family 2 subfamily B member 6	NM_000767.5	TGGCCGGGGAAAAATCGCCA	GAAGAGCTCAAACAGCTGGCCGAA	373	60	(Huch et al., 2015)
	CYP2C8	Cytochrome P450 family 2 subfamily C member 8	NM_000770.3	CCGTGTTCAAGAGGAAGCTC	AGTGGGATCACAGGGTGAAG	73	60	Primer3
	CYP2C9	Cytochrome P450 family 2 subfamily C member 9	NM_000771.4	CCTCTGGGGCATTATCCATC	ATATTTGCACAGTGAAACATAGGA	137	60	(Sa-ngiamsuntorn et al., 2011)
	CYP2C19	Cytochrome P450 family 2 subfamily C member 19	NM_000769.4	CAACAACCCTCGGGACTTTA	GTCTCTGTCCCAGCTCCAAG	132	64	(Chen et al., 2014a)
	CYP2D6	Cytochrome P450 family 2 subfamily D member 6	NM_000106.6	CTAAGGGAACGACACTCATCAC	CTCACCAGGAAAGCAAAGACAC	289	60	(Hara and Adachi, 2002)
	CYP2E1	Cytochrome P450 family 2 subfamily E member 1	NM_000773.3	GCAAGAGATGCCCTACATGGA	GGGCACGAGGGTGATGAA	64	62	(Chen et al., 2014b)

	CYP3A4	Cytochrome P450 family 3 subfamily A member 4	NM_017460.5	GAAACACAGATCCCCCTGAA	CTGGTGTTCTCAGGCACAGA	161	62	(Ryu et al., 2018)
	CYP24A1	Cytochrome P450 family 24 subfamily A member 1	NM_000782.4	GGCAACAGTTCTGGGTGAAT	TATTTGCGGACAATCCAACA	249	60	Primer3
	CES2	Carboxylesterase 2	NM_001365405.1	CATGTTTGTGATCCCTGCAC	AGTTGCCCCCAAAGAAACTT	176	64	Primer3
	MAOA	Monoamine oxidase A	NM_000240.3	CACACCTTTTGGGAAATGCT	TGGACTGGCATTCATTTTGA	204	60	Primer3
	NAT	N-acetyltransferase 2	XM_017012938.1	ACGTCTCCAACATCTTCATTTATAACC	TCAACCTCTTCCTCAGTGAGAGTTTTA	161	60	(Gonzalez-Arias et al., 2015)
Oxidative stress indicators	*Catalase*	Catalase	NM_001752.3	GAACTGTCCCTACCGTGCTCGA	CCAGAATATTGGATGCTGTGCTCCAGG	156	62	(Thanuthanakhun et al., 2017)
	PXR	Nuclear receptor subfamily 1 group I member 2	NM_003889.3	CTGGAGGTGAGACCCAAAGA	CACATACACGGCAGATTTGG	133	60	(Mencarelli et al., 2010)
	SOD1	Superoxide dismutase 1	NM_000454.4	CTGAAGGCCTGCATGGATTC	CCAAGTCTCCAACATGCCTCTC	186	60	(Ryu et al., 2018)
	GPx1	Glutathione peroxidase 1	NM_000581.3	TGGCTTCTTGGACAATTGCG	CCACCAGGAACTTCTCAAAG	530	54	(Ryu et al., 2018)
	HO1	Heme oxygenase 1	NM_002133.2	CTCAAACCTCCAAAAGCC	TCAAAAACCACCCCAACCC	220	62	(Ryu et al., 2018)
	iNOS	Nitric oxide synthase 2	NM_000625.4	CTCTATGTTTGCGGGGATGT	TTCTTCGCCTCGTAAGGAAA	179	60	(Ryu et al., 2018)

	Bad	BCL2 associated agonist of cell death	NM_004322.3	CCCAGAGTTTGAGCCGAGTG	CCCATCCCTTCGTCGTCCT	249	60	(Wang et al., 2015)
	Bax	BCL2 associated X, apoptosis regulator	NM_001291428.1	GTCAGCTGCCACTCGGAAA	AGTAACATGGAGCTGCAGAGGAT	81	60	(Riwaldt et al., 2017)
	Bcl-2	BCL2, apoptosis regulator	NM_000633.2	TCAGAGACAGCCAGGAGAAATCA	CCTGTGGATGACTGAGTACCTGAA	131	60	(Kopp et al., 2018)
	Bcl-XL	BCL2 like 1	NM_138578.2	ATGGCAGCAGTAAAGCAAGC	CGGAAGAGTTCATTCACTACCTGT	149	60	(Kehn-Hall et al., 2012)
	Bid	BH3 interacting domain death agonist	NM_197966.2	ACTGGTGTTTGGCTTCCTCC	ATTCTTCCCAAGCGGGAGTG	159	60	(Li et al., 2016b)
Apoptosis-related genes	*Casp3*	Caspase 3	NM_001354777.1	AACTGCTCCTTTTGCTGTGATCT	GCAGCAAACCTCAGGGAAAC	130	60	(Kopp et al., 2016)
	Casp7	Caspase 7	XM_017016763.1	AGTGACAGGTATGGGCGTTCG	GCATCTATCCCCCCTAAAGTGG	274	60	(Ye et al., 2012)
	Casp8	Caspase 8	XM_005246885.2	CATCCAGTCACTTTGCCAGA	GCATCTGTTTCCCCATGTTT	128	62	(Wang et al., 2017)
	Casp9	Caspase 9	XM_005246014.2	TTCCCAGGTTTTGTTTCCTG	CCTTTCACCGAAACAGCATT	143	60	(Cheng et al., 2017)
	Fas	Fas cell surface death receptor	NM_000043.5	AGTTGGGGAAGCTCTTTCACTT	CAGTCTTCCTCAATTCCAATCC	163	60	(Zhang et al., 2016)
	PUMA	BCL2 binding component 3	NM_001127240.2	GGGGGACTTTCTCTGCACCA	CCGTCCCTCTCCTGGCTTCT	157	60	Primer3
	TGF-β1	Transforming growth	NM_000660.5	CACGTGGAGCTGTACCAGAA	CAGCCGGTTGCTGAGGTA	60	60	(Howe et al., 2017)

		factor, beta 1						
	p53	Tumor protein p53	NM_000546.5	CCCAAGCAATGGATGATTTGA	GGCATTCTGGGAGCTTCATCT	91	62	(Ryu et al., 2018)
	APC	APC, WNT signaling pathway regulator	NM_000038.5	CTGTCCTGCTGTGTGTGTTC	TGCTTTCACACTTCCAACTTCT	376	60	Primer3
Inflammation-related genes	*IL-1β*	Interleukin 1-beta	M15330.1	ACAGATGAAGTGCTCCTTCCA	GTCGGAGATTCGTAGCTGGAT	73	60	(Ryu et al., 2018)
	IL1RN	Interleukin 1 receptor antagonist	NM_000577.4	GAGCTTCTGGCACTTGGAGACT	TAGGGAACTTTGCACCCAACAT	195	60	(Huang et al., 2013)
	IL-6	Interleukin 6	NM_000600.4	GTAGCCGCCCCACACAGA	CATGTCTCCTTTCTCAGGGCTG	101	66	(Nhu et al., 2010)
	NF-κB	Nuclear factor kappa-B DNA binding subunit	M58603.1	CCTCTGTGTTTGTCCAGCT	CCGAAAAATTGGGCATGAGC	135	60	(Ryu et al., 2018)
	TNF	Tumor necrosis factor	NM_000594.3	ACAAGCCTGTAGCCCATGTT	AAAGTAGACCTGCCCAGACT	428	66	(Ryu et al., 2018)
	TLR2	Toll like receptor 2	NM_001318789.1	ATCCTCCAATCAGGCTTCTCT	ACACCTCTGTAGGTCACTGTTG	163	61	(Ryu et al., 2018)
	TLR4	Toll like receptor 4	NM_003266.3	TGGAAGTTGAACGAATGGAATGTG	ACCAGAACTGCTACAACAGATACT	147	60	(Yan et al., 2017)
Tight junction structure-related genes	*OCLN*	Occludin	NM_001205255.1	TCAGGGAATATCCACCTATCACTTCAG	CATCAGCAGCAGCCATGTACTCTTCAC	189	64	(Ryu et al., 2018)
	CLDN1	Claudin 1	NM_021101.4	CGATGAGGTGCAGAAGATGA	CCAGTGAAGAGAGCCTGACC	174	65	(Raleigh et al., 2010)

	CLDN3	Claudin 3	NM_001306.3	CCAACCTGCATGGACTGTGA	TCGACGGGGTGGTCAAGTAT	80	60	(Becker et al., 2018)
	VIL1	Villin 1	NM_007127.2	CAAGACAGGCTCACTCACCA	TGTCATAGGACAGGCTGCTG	203	60	Primer3
	TJP1	Tight junction protein 1	NM_001301025.2	CTCACCACAAGCGCAGCCACAA	ACAGCAGAGGTTGATGATGCTGGG	141	65	(Devriese et al., 2017)
Reference gene	*ACTB*	Actin beta	NM_001101.3	CATCGAGCACGGCATCGTCA	TAGCACAGCCTGGATAGCAAC	211	64	(Ryu et al., 2018)
**Rat**								
Metabolism-related genes	*Cyp1a2*	Cytochrome P450, family 1, subfamily a, polypeptide 2	NM_012541.3	AGGGACACCTCACTGAATGG	CCGAAGAGCATCACCTTCTC	182	60	(Ma et al., 2016)
	Cyp2b1	Cytochrome P450, family 2, subfamily b, polypeptide 1	NM_001134844.1	TGAGAACCTCATGATCTCCCTGC	AGGAAACCATAGCGGAGTGTGG	81	60	(Zhang et al., 2018)
	Cyp2c7	Cytochrome P450, family 2, subfamily c, polypeptide 7	NM_017158.2	ACGGGGAGAAGTTTTCTGGT	TGTGCTTCCTCTTGAACACG	181	60	Primer3
	Cyp2c11	Cytochrome P450, subfamily 2, polypeptide 11	NM_019184.2	AAAAGCACAATCCGCAGTCT	GCATCTGGCTCCTGTCTTTC	216	60	(Ma et al., 2016)
	Cyp2c6v1	Cytochrome P450, family 2, subfamily C, polypeptide 6,	NM_001013904.1	ATTGTCCGGGAAGTCATACG	TGTTGTCTCTGTCCCAGCAG	233	60	Primer3

		variant 1						
	Cyp2d3	Cytochrome P450, family 2, subfamily d, polypeptide 3	NM_173093.1	ATGAGGTCATAGGGCAGGTG	TCAGCACTGAGGACAGGTTG	204	60	Primer3
	Cyp2e1	Cytochrome P450, family 2, subfamily e, polypeptide 1	NM_031543.1	CCTACATGGATGCTGTGGTG	CTGGAAACTCATGGCTGTCA	171	60	(Beltran-Ramirez et al., 2010)
	Cyp3a2	Cytochrome P450, family 3, subfamily a, polypeptide 2	NM_153312.2	AGTGGGGATTATGGGGAAAG	CTCCAAATGATGTGCTGGTG	242	60	(Ma et al., 2016)
	Cyp24a1	Cytochrome P450, family 24, subfamily a, polypeptide 1	NM_201635.3	GCATGGATGAGCTGTGCGA	AATGGTGTCCCAAGCCAGC	269	60	(Durk et al., 2012)
	CES2	Carboxylesterase 2H	NM_001044258.2	GTTGGCCTCTGCTGACTTTC	ATTCAGGAGGCCACATCATC	176	60	Primer3
	MAOA	Monoamine oxidase A	NM_033653.1	GCCAAAGTTCTGGGATCTCAAGAAGC	CACCAGTGATCTTGAGCAGACCAG	388	64	(Zhu et al., 2012)
	NAT	N-acetyltransferase 1	NM_001037315.1	GAGGCCACTTTTGACCACAT	AAGGTGGACCATTTCACTGC	168	60	Primer3
Oxidative stress indicators	*Catalase*	Catalase	NM_012520.2	ACAACTCCCAGAAGCCTAAGAATG	GCTTTTCCCTTGGCAGCTATG	76	60	(Chen et al., 2006)
	PXR	Nuclear receptor subfamily 1, group I, member 2	NM_052980.2	GCAGCTGCGCGGAGAA	TTTCCCGTCGCTCTTGGA	64	60	(Hartley et al., 2004)

	SOD1	Superoxide dismutase 1	NM_017050.1	ACACAAGGCTGTACCACTGC	CCACATTGCCCAGGTCTCC	103	60	(Ryu et al., 2018)
	GPx1	Glutathione peroxidase 1	NM_030826.4	GTCCACCGTGTATGCCTTCTCC	TCTCCTGATGTCCGAACTGATTGC	218	66	(Ryu et al., 2018)
	HO1	Heme oxygenase 1	NM_012580.2	CACGCATATACCCGCTACCT	AAGGCGGTCTTAGCCTCTTC	227	66	(Ryu et al., 2018)
	iNOS	Inducible nitric oxide synthase	AY211532.1	CACCACCCTCCTTGTTCAAC	CAATCCACAACTCGCTCCAA	132	60	(Ryu et al., 2018)
Apoptosis-related genes	*Bad*	BCL2-associated agonist of cell death	NM_022698.1	CAGGCAGCCAATAACAGT	CCATCCCTTCATCTTCCTC	100	60	(Melgar-Rojas et al., 2015)
	Bax	BCL2 associated X, apoptosis regulator	U49729.1	AGGGTGGCTGGGAAGGC	TGAGCGAGGCGGTGAGG	93	66	(Ryu et al., 2018)
	Bcl-2	BCL2, apoptosis regulator	NM_016993.1	ATCGCTCTGTGGATGACTGAGTAC	AGAGACAGCCAGGAGAAATCAAAC	134	60	(Ryu et al., 2018)
	Bcl-XL	Bcl2-like 1	NM_001033670.1	GGTCGCATTGTGGCCTTCTT	CTCTCGGCTGCTGCATTGTT	197	60	(Produit-Zengaffinen et al., 2009)
	Bid	BH3 interacting domain death agonist	NM_022684.1	CGACGAGGTGAAGACATCCT	AGCAGAGATGGTGCATGACT	108	60	(Li et al., 2016b)
	Casp3	Caspase 3	NM_012922.2	AGTCTGACTGGAAAGCCGAAA	TCTGTCTCAATACCGCAGTCC	77	60	Primer3
	Casp7	Caspase 7	NM_022260.3	CAACGACACCGACGCTAATC	GGTCCTTGCCATGCTCATTC	161	60	(Almutiri et al., 2018)
	Casp8	Caspase 8	NM_0222	CTGGGA	CATGTCC	117	65	(Ruan

			77.1	AGGATCG ACGATTA	TGCATTT TGATGG			et al., 2015)
	Casp9	Caspase 9	NM_031632.1	AGCCAGA TGCTGTC CCATAC	CAGGAG ACAAAA CCTGGGA A	124	60	(Ryu et al., 2018)
	Fas	Fas cell surface death receptor	NM_139194.2	ACCTGGT GACCCTG AATCTG	TGATACC AGCACTG GAGCAG	231	60	Primer3
	PUMA	Bcl-2 binding component 3	NM_173837.2	CTGGAGC CCCAGA AATGGAG	AGGGTCC CCCAAGT CCGTAT	236	63	Primer3
	TGF-β1	Transforming growth factor, beta 1	NM_021578.2	ATGACAT GAACCG ACCCTTC	ACTTCCA ACCCAG GTCCTTC	177	66	(Li et al., 2016a)
	p53	Tumor protein p53	NM_030989.3	GTTCCGA GAGCTG AATGAGG	TTTTATG GCGGGA CGTAGAC	125	66	(Ryu et al., 2018)
	APC	APC, WNT signaling pathway regulator	NM_012499.1	AAACGA GCACAG CGAAGA AT	GCTTTCT GCCACTC CTTGAC	174	60	Primer3
Inflammation-related genes	*IL-1β*	Interleukin 1 beta	NM_031512.2	CTTGTCG AGAATGG GCAGTCT	TGTGCCA CGGTTTT CTTATGG	85	62	(Ryu et al., 2018)
	IL1RN	Interleukin 1 receptor antagonist	NM_022194.2	CTTATTG CCTCTGC CCTCTG	TGATTGG TCTGGAC TGTGGA	208	62	Primer3
	IL-6	Interleukin 6	NM_012589.2	TCCTACC CCAACTT CCAATGC TC	TTGGATG GTCTTGG TCCTTAG CC	79	66	(Peinnequin et al., 2004)
	NF-κB	Nuclear factor kappa B subunit 1	NM_001276711	CTTCTCG GAGTCCC TCACTG	CCAATAG CAGCTGG AAAAGC	366	60	(Ryu et al., 2018)
	TNF	Tumor necrosis factor-alpha	X66539.1	TCGTAGC AAACCA CCAAGC A	CCCTTGA AGAGAA CCTGGGA GTA	151	60	(Ryu et al., 2018)
	TLR2	Toll-like	NM_1987	CGCTTCC	GGTTGTC	286	66	(Ryu et

		receptor 2	69.2	TGAACTTGTCC	ACCTGCTTCCA			al., 2018)
	TLR4	Toll-like receptor 4	NM_019178.1	GATTGCTCAGACATGGCAGTTTC	CACTCGAGGTAGGTGTTTCTGCTAA	135	66	(Miao et al., 2011)
Tight junction structure-related genes	*OCLN*	Occludin	NM_031329.2	AGTACATGGCTGCTGCTGATG	CCCACCATCCTCTTGATGTGT	127	60	(Ryu et al., 2018)
	CLDN1	Claudin 1	NM_031699.2	CACTTCCAGACTCCACCACC	CCAGGAGGTTAGCGCTGATA	92	62	Primer3
	CLDN3	Claudin 3	NM_031700.2	TTTCTCCCATGGTGAAGAGG	TCTGAGACTGGGTTGGCTCT	154	60	Primer3
	VIL1	Villin 1	NM_001108224.2	GCTCTTTGAGTGCTCCAACC	GGGGTGGGTCTTGAGGTATT	199	62	(Khan et al., 2009)
	TJP1	Tight junction protein 1	NM_001106266.1	GCACAGCAATGGAGGAAACA	CCCACTTTTCCTTAGCTGCTG	269	62	Primer3
Reference gene	*Actb*	Actin, beta	NM_031144.3	AACCTTCTTGCAGCTCCTCCG	CCATACCCACCATCACACCCT	193	60	(Li et al., 2015)

References

Almutiri, S., Berry, M., Logan, A., and Ahmed, Z. (2018). Non-viral-mediated suppression of AMIGO3 promotes disinhibited NT3-mediated regeneration of spinal cord dorsal column axons. Sci Rep 8(1), 10707. doi: 10.1038/s41598-018-29124-z.

Becker, A., Leskau, M., Schlingmann-Molina, B.L., Hohmeier, S.C., Alnajjar, S., Escobar, H.M., et al. (2018). Functionalization of gold-nanoparticles by the Clostridium perfringens enterotoxin C-terminus for tumor cell ablation using the gold nanoparticle-mediated laser perforation technique. Sci Rep 8(1), 14963. doi: 10.1038/s41598-018-33392-0.

Beltran-Ramirez, O., Sokol, S., Le-Berre, V., Francois, J.M., and Villa-Trevino, S. (2010). An approach to the study of gene expression in hepatocarcinogenesis initiation. Transl Oncol 3(2), 142-148.

Chen, H., Shen, Z.Y., Xu, W., Fan, T.Y., Li, J., Lu, Y.F., et al. (2014a). Expression of P450 and nuclear receptors in normal and end-stage Chinese livers. World J Gastroenterol 20(26), 8681-8690. doi: 10.3748/wjg.v20.i26.8681.

Chen, H., Zhang, X., Feng, Y., Rui, W., Shi, Z., and Wu, L. (2014b). Bioactive components of Glycyrrhiza uralensis mediate drug functions and properties through regulation of CYP450 enzymes. Mol Med Rep 10(3), 1355-1362. doi: 10.3892/mmr.2014.2331.

Chen, J., Rider, D.A., and Ruan, R. (2006). Identification of valid housekeeping genes and antioxidant enzyme gene expression change in the aging rat liver. J Gerontol A Biol Sci Med Sci 61(1), 20-27.

Cheng, Y.M., Shen, C.J., Chang, C.C., Chou, C.Y., Tsai, C.C., and Hsu, Y.C. (2017). Inducement of apoptosis by cucurbitacin E, a tetracyclic triterpenes, through death receptor 5 in human cervical cancer cell lines. Cell Death Discov 3, 17014. doi: 10.1038/cddiscovery.2017.14.

Devriese, S., Eeckhaut, V., Geirnaert, A., Van den Bossche, L., Hindryckx, P., Van de Wiele, T., et al. (2017). Reduced Mucosa-associated Butyricicoccus Activity in Patients with Ulcerative Colitis Correlates with Aberrant Claudin-1 Expression. J Crohns Colitis 11(2), 229-236. doi: 10.1093/ecco-jcc/jjw142.

Durk, M.R., Chan, G.N., Campos, C.R., Peart, J.C., Chow, E.C., Lee, E., et al. (2012). 1alpha,25-Dihydroxyvitamin D3-liganded vitamin D receptor increases expression and transport activity of P-glycoprotein in isolated rat brain capillaries and human and rat brain microvessel endothelial cells. J Neurochem 123(6), 944-953. doi: 10.1111/jnc.12041.

Gonzalez-Arias, C.A., Crespo-Sempere, A., Marin, S., Sanchis, V., and Ramos, A.J. (2015). Modulation of the xenobiotic transformation system and inflammatory response by ochratoxin A exposure using a co-culture system of Caco-2 and HepG2 cells. Food Chem Toxicol 86, 245-252. doi: 10.1016/j.fct.2015.10.007.

Hara, H., and Adachi, T. (2002). Contribution of hepatocyte nuclear factor-4 to down-regulation of CYP2D6 gene expression by nitric oxide. Mol Pharmacol 61(1), 194-200.

Hartley, D.P., Dai, X., He, Y.D., Carlini, E.J., Wang, B., Huskey, S.E., et al. (2004). Activators of the rat pregnane X receptor differentially modulate hepatic and intestinal gene expression. Mol Pharmacol 65(5), 1159-1171. doi: 10.1124/mol.65.5.1159.

Howe, G.A., Kazda, K., and Addison, C.L. (2017). MicroRNA-30b controls endothelial cell capillary morphogenesis through regulation of transforming growth factor beta 2. PLoS One 12(10), e0185619. doi: 10.1371/journal.pone.0185619.

Huang, S., Liu, F., Niu, Q., Li, Y., Liu, C., Zhang, L., et al. (2013). GLIPR-2 overexpression

in HK-2 cells promotes cell EMT and migration through ERK1/2 activation. PLoS One 8(3), e58574. doi: 10.1371/journal.pone.0058574.

Huch, M., Gehart, H., van Boxtel, R., Hamer, K., Blokzijl, F., Verstegen, M.M., et al. (2015). Long-term culture of genome-stable bipotent stem cells from adult human liver. Cell 160(1-2), 299-312. doi: 10.1016/j.cell.2014.11.050.

Kehn-Hall, K., Narayanan, A., Lundberg, L., Sampey, G., Pinkham, C., Guendel, I., et al. (2012). Modulation of GSK-3beta activity in Venezuelan equine encephalitis virus infection. PLoS One 7(4), e34761. doi: 10.1371/journal.pone.0034761.

Khan, A.A., Chow, E.C., Porte, R.J., Pang, K.S., and Groothuis, G.M. (2009). Expression and regulation of the bile acid transporter, OSTalpha-OSTbeta in rat and human intestine and liver. Biopharm Drug Dispos 30(5), 241-258. doi: 10.1002/bdd.663.

Kopp, S., Sahana, J., Islam, T., Petersen, A.G., Bauer, J., Corydon, T.J., et al. (2018). The role of NFkappaB in spheroid formation of human breast cancer cells cultured on the Random Positioning Machine. Sci Rep 8(1), 921. doi: 10.1038/s41598-017-18556-8.

Kopp, S., Slumstrup, L., Corydon, T.J., Sahana, J., Aleshcheva, G., Islam, T., et al. (2016). Identifications of novel mechanisms in breast cancer cells involving duct-like multicellular spheroid formation after exposure to the Random Positioning Machine. Sci Rep 6, 26887. doi: 10.1038/srep26887.

Li, C.W., Wang, Q., Li, J., Hu, M., Shi, S.J., Li, Z.W., et al. (2016a). Silver nanoparticles/chitosan oligosaccharide/poly(vinyl alcohol) nanofiber promotes wound healing by activating TGFbeta1/Smad signaling pathway. Int J Nanomedicine 11, 373-386. doi: 10.2147/IJN.S91975.

Li, K., Li, Y., Ma, Z., and Zhao, J. (2015). Crocin exerts anti-inflammatory and anti-catabolic effects on rat intervertebral discs by suppressing the activation of JNK. Int J Mol Med 36(5), 1291-1299. doi: 10.3892/ijmm.2015.2359.

Li, X., Zhao, Y., Xia, Q., Zheng, L., Liu, L., Zhao, B., et al. (2016b). Nuclear translocation of annexin 1 following oxygen-glucose deprivation-reperfusion induces apoptosis by regulating Bid expression via p53 binding. Cell Death Dis 7(9), e2356. doi: 10.1038/cddis.2016.259.

Ma, L.L., Wu, Z.T., Wang, L., Zhang, X.F., Wang, J., Chen, C., et al. (2016). Inhibition of hepatic cytochrome P450 enzymes and sodium/bile acid cotransporter exacerbates leflunomide-induced hepatotoxicity. Acta Pharmacol Sin 37(3), 415-424. doi: 10.1038/aps.2015.157.

Melgar-Rojas, P., Alvarado, J.C., Fuentes-Santamaria, V., Gabaldon-Ull, M.C., and Juiz, J.M. (2015). Validation of Reference Genes for RT-qPCR Analysis in Noise-Induced Hearing Loss: A Study in Wistar Rat. PLoS One 10(9), e0138027. doi: 10.1371/journal.pone.0138027.

Mencarelli, A., Migliorati, M., Barbanti, M., Cipriani, S., Palladino, G., Distrutti, E., et al. (2010). Pregnane-X-receptor mediates the anti-inflammatory activities of rifaximin on detoxification pathways in intestinal epithelial cells. Biochem Pharmacol 80(11), 1700-1707. doi: 10.1016/j.bcp.2010.08.022.

Miao, J., Zheng, L., Zhang, J., Ma, Z., Zhu, W., and Zou, S. (2011). The effect of taurine on the toll-like receptors/nuclear factor kappa B (TLRs/NF-kappaB) signaling pathway in Streptococcus uberis-induced mastitis in rats. Int Immunopharmacol 11(11), 1740-1746. doi: 10.1016/j.intimp.2011.06.008.

Nhu, Q.M., Shirey, K., Teijaro, J.R., Farber, D.L., Netzel-Arnett, S., Antalis, T.M., et al. (2010). Novel signaling interactions between proteinase-activated receptor 2 and Toll-like receptors in vitro and in vivo. Mucosal Immunol 3(1), 29-39. doi:

10.1038/mi.2009.120.

Peinnequin, A., Mouret, C., Birot, O., Alonso, A., Mathieu, J., Clarencon, D., et al. (2004). Rat pro-inflammatory cytokine and cytokine related mRNA quantification by real-time polymerase chain reaction using SYBR green. BMC Immunol 5, 3. doi: 10.1186/1471-2172-5-3.

Produit-Zengaffinen, N., Pournaras, C.J., and Schorderet, D.F. (2009). Retinal ischemia-induced apoptosis is associated with alteration in Bax and Bcl-x(L) expression rather than modifications in Bak and Bcl-2. Mol Vis 15, 2101-2110.

Raleigh, D.R., Marchiando, A.M., Zhang, Y., Shen, L., Sasaki, H., Wang, Y., et al. (2010). Tight junction-associated MARVEL proteins marveld3, tricellulin, and occludin have distinct but overlapping functions. Mol Biol Cell 21(7), 1200-1213. doi: 10.1091/mbc.E09-08-0734.

Riwaldt, S., Monici, M., Graver Petersen, A., Birk Jensen, U., Evert, K., Pantalone, D., et al. (2017). Preparation of A Spaceflight: Apoptosis Search in Sutured Wound Healing Models. Int J Mol Sci 18(12). doi: 10.3390/ijms18122604.

Ruan, Y., Hu, K., and Chen, H. (2015). Autophagy inhibition enhances isorhamnetininduced mitochondriadependent apoptosis in nonsmall cell lung cancer cells. Mol Med Rep 12(4), 5796-5806. doi: 10.3892/mmr.2015.4148.

Ryu, B., Kim, C.Y., Oh, H., Kim, U., Kim, J., Jung, C.R., et al. (2018). Development of an alternative zebrafish model for drug-induced intestinal toxicity. J Appl Toxicol 38(2), 259-273. doi: 10.1002/jat.3520.

Sa-ngiamsuntorn, K., Wongkajornsilp, A., Kasetsinsombat, K., Duangsa-ard, S., Nuntakarn, L., Borwornpinyo, S., et al. (2011). Upregulation of CYP 450s expression of immortalized hepatocyte-like cells derived from mesenchymal stem cells by enzyme inducers. BMC Biotechnol 11, 89. doi: 10.1186/1472-6750-11-89.

Thanuthanakhun, N., Nuntakarn, L., Sampattavanich, S., Anurathapan, U., Phuphanitcharoenkun, S., Pornpaiboonstid, S., et al. (2017). Investigation of FoxO3 dynamics during erythroblast development in beta-thalassemia major. PLoS One 12(11), e0187610. doi: 10.1371/journal.pone.0187610.

Wang, G., Chen, H., and Liu, J. (2015). The long noncoding RNA LINC01207 promotes proliferation of lung adenocarcinoma. Am J Cancer Res 5(10), 3162-3173.

Wang, G., Zhang, M., Li, Y., Zhou, J., and Chen, L. (2017). Studying the Effect of Downregulating Autophagy-Related Gene LC3 on TLR3 Apoptotic Pathway Mediated by dsRNA in Hepatocellular Carcinoma Cells. Cancer Res Treat 49(1), 230-245. doi: 10.4143/crt.2015.506.

Yan, C., Li, B., Fan, F., Du, Y., Ma, R., Cheng, X.D., et al. (2017). The roles of Toll-like receptor 4 in the pathogenesis of pathogen-associated biliary fibrosis caused by Clonorchis sinensis. Sci Rep 7(1), 3909. doi: 10.1038/s41598-017-04018-8.

Ye, Y., Xiao, Y., Wang, W., Gao, J.X., Yearsley, K., Yan, Q., et al. (2012). Singular v dual inhibition of SNF2L and its isoform, SNF2LT, have similar effects on DNA damage but opposite effects on the DNA damage response, cancer cell growth arrest and apoptosis. Oncotarget 3(4), 475-489. doi: 10.18632/oncotarget.479.

Zhang, D., Li, Y., Wang, R., Li, Y., Shi, P., Kan, Z., et al. (2016). Inhibition of REST Suppresses Proliferation and Migration in Glioblastoma Cells. Int J Mol Sci 17(5). doi: 10.3390/ijms17050664.

Zhang, P., Yang, M., Zeng, L., and Liu, C. (2018). P38/TRHr-Dependent Regulation of TPO in Thyroid Cells Contributes to the Hypothyroidism of Triclosan-Treated Rats. Cell Physiol Biochem 45(4), 1303-1315. doi: 10.1159/000487558.

Zhu, K.Y., Mao, Q.Q., Ip, S.P., Choi, R.C., Dong, T.T., Lau, D.T., et al. (2012). A standardized chinese herbal decoction, kai-xin-san, restores decreased levels of neurotransmitters and neurotrophic factors in the brain of chronic stress-induced depressive rats. Evid Based Complement Alternat Med 2012, 149256. doi: 10.1155/2012/149256.
